# Cladistic analysis of the genus *Bruggmanniella* Tavares (Diptera, Cecicomyiidae, Asphondyliini) with evolutionary inferences on the gall inducer-host plant association and description of a new Brazilian species

**DOI:** 10.1371/journal.pone.0227853

**Published:** 2020-02-05

**Authors:** Carolina De Almeida Garcia, Carlos José Einicker Lamas, Maria Virginia Urso-Guimarães

**Affiliations:** 1 Laboratório de Diptera, Museu de Zoologia da Universidade de São Paulo, São Paulo, São Paulo, Brazil; 2 Departamento de Biologia, Laboratório de Sistemática de Diptera, Universidade Federal de São Carlos, Sorocaba, São Paulo, Brazil; Universita degli Studi di Roma La Sapienza, ITALY

## Abstract

In this study, we present a phylogenetic analysis of the genus *Bruggmanniella* Tavares based on morphological features. Cladistic analyses were conducted using 57 characters from 26 species. All species of *Bruggmanniella* except for *B*. *byrsonimae* were selected as ingroup and the genera *Asphondylia* Loew, *Bruggmannia* Tavares, *Illiciomyia* Tokuda, *Parazalepidota* Maia, *Pseudasphondylia* Monzen, *Schizomyia* Kieffer, and *Lopesia* Rübsaamen as outgroup. We used characters from larvae, pupae, adults, and galls. The results of this study supported *Bruggmanniella* as the sister group of *Pseudasphondylia*. *Bruggmanniella actinodaphnes* Tokuda and Yukawa and *B*. *cinnamomi* Tokuda and Yukawa have been moved to genus *Pseudasphondylia* (*Pseudasphondylia actinodaphnes* (Tokuda and Yukawa) **comb. nov.** and *Pseudasphondylia cinnamomi* (Tokuda and Yukawa) **comb. nov.**). The new genus *Odontokeros* **gen. nov.** has been erected for the single species *Odontokeros brevipes* (Lin, Yang & Tokuda) **comb. nov.** In addition, we described a new Brazilian species, *Bruggmanniella miconia* Garcia, Lamas and Urso-Guimarães **sp. nov.** Identification keys to the New World species of *Bruggmanniella* are presented.

## Introduction

The family Cecidomyiidae is one of the most diversified families of Diptera, with more than 6,600 described species worldwide [1]. Estimates indicate thousands [2, 3] to more than a million [4] of undescribed species. The family belongs to the Sciaroidea within the infraorder Bibionomorpha [5, 6] and the monophyly is recognized without controversy [7, 8]. The phylogenetic analyses of Sikora et al. [9] and Dorchin et al. [10] corroborate the classification of the main branches of Cecidomyiidae outlined in Gagné and Jaschhof [1].

*Bruggmanniella* Tavares, 1909 is a genus of the tribe Asphondyliini with 13 valid species. Asphondyliini can be recognized by the cylindrical antennal flagellomeres of both sexes, the conspicuously long seventh sternite of the female abdomen, short gonostylus located dorsally on gonocoxite, and larvae and pupae with strong and sclerotized structures [10, 11].

*Bruggmanniella* has 11 known species, eight with distribution in the Neotropical Region, three species in the Oriental/Palearctic regions and one in the Nearctic [1, 12]. They are recognized by the presence of a divided tooth on the gonostyle. The pupa has well developed antennal horns, absence of frontal horns and protuberant abdominal spiracles. The larva has four teeth, inners larger than outer ones.

Among herbivorous insects, cecidomyiids are well known for their remarkable ability to induce galls on various plant taxa [13–15]. Galls variety and complexity have been regarded as an “extended phenotype” of gall inducers in some phylogenetic studies [16]. These insects have a high capacity of host shifting which led them to colonize a huge range of genera and plant species [17]. All the New World species of *Bruggmanniella* induce stem or ovary/fruits galls on species of nine different families of plants–Anacardiaceae, Annonaceae, Celastraceae, Dilleniaceae, Fabaceae, Lauraceae, Malpighiaceae, Moraceae, and Sapotaceae [1]–suggesting more affinity with the infested organ than with the phylogenetically related host plant species. On the other hand, most Oriental and Eastern Palearctic species of *Pseudasphondylia* Monzen, *B*. *brevipes* Lin, Tokuda & Yang, and *Illiciomyia* Tokuda induce leaf galls on species of Lauraceae.

Some authors have been considered *Bruggmanniella* Tavares as a genus closely related to *Pseudasphondylia* and *Illyciomyia* [1, 16, 18–25]. Similarities between these genera are based on the presence of a divided tooth on gonostyle of male terminalia [19, 25]. Tokuda and Yukawa [21–23] also discuss such relation and suggest more morphological and phylogenetic studies to determine the relationship among these genera.

In this study, we investigate i) the phylogenetic relationship of all species of *Bruggmanniella* and the available *Pseudasphondylia* species through the study of morphological characters of adult and immature stages; ii) the evolution of the gall maker and the host plant association. In addition, we describe a new genus, *Odontokerus* Garcia, Lamas and Urso-Guimarães **gen. nov.**, and a new species, *Bruggmanniella miconia* Garcia, Lamas and Urso-Guimarães **sp. nov.**

*Bruggmanniella miconia* **sp. nov.** was reared from globoid stem galls of *Miconia* cf. *cinnamomifolia* (Melastomataceae) from São Paulo State, Brazil. *Miconia* Ruiz and Pavón is an endemic genus to the Neotropical Region and the largest one of woody flowering plants with about 1,070 species [26, 27]. There is only one record of galls on buds of *Miconia cinnamomifolia* induced by *Epihormomyia miconiae* Maia from the state of Rio de Janeiro, Brazil [28]. The new species is described, illustrated and compared with its nine Neotropical congeners. Identification keys to larvae, pupae, and adults of all species are also provided herein.

## Material and methods

### Collection and preparation of material

Branches of *Miconia* cf. *cinnamomifolia* (Melastomataceae) were collected from an area of Semideciduous Seasonal Forest on the Universidade Federal de São Carlos (UFSCar), *campus* Sorocaba (23°35'09.4" S; 47°30'59.9" W), from 2015 to 2019. The botanical material was collected under collecting permit number 17635–1 SISBIO/IBAMA. The sampled location reported in this work is not a protected area and the field studies did not involve endangered or protected species.

Attempts to rear adults from galls began since the first foray into the sampled area. In the laboratory, some of the galls were kept in plastic pots for rearing the adults, but no male adults were obtained, only pupae in the last stage of development. Other galls were dissected under a stereomicroscope to obtain immature instars (larva and pupa). The obtained specimens were mounted on slides according to the methodology outlined in Gagné [11]. Additional material was stored in 100% ethanol for DNA extraction. Morphological terminology for adults follows Cumming and Wood [29] and immature terminology follows Gagné [11].

Types of the new species are deposited in the Museu de Zoologia of the Universidade de São Paulo, São Paulo, Brazil (MZSP). Voucher specimens of the host plant species are deposited in the Herbarium of the Universidade Federal de São Carlos, *campus* Sorocaba, Brazil.

### DNA extraction

DNA was extracted from two whole pupae using DNeasy Blood & Tissue Kit (Quiagen) following the manufacturer’s instructions. The mitochondrial cytochrome oxidase subunit I (COI) barcoding region was amplified by polymerase chain reaction (PCR) using the following primers: LCO1490 (forward) and HCO2198 (reverse) [30]. The PCR products were purified using Exo/SAP and the COI sequences were assembled and aligned using the Geneious Prime 2020 software [31]. The COI-alignment was constructed using the MUSCLE algorithm in Geneious with eight iterations. The obtained sequences were deposited in the GenBank under the accession number MN686345.

### Selection of taxa

For the phylogenetic study, a total of 26 species were selected and examined. Fifteen species were selected as outgroups, including the genera *Asphondylia* Loew, *Bruggmannia* Tavares, *Illiciomyia* Tokuda, *Parazalepidota* Maia, *Pseudasphondylia* Monzen, *Schizomyia* Kieffer (Asphondyliini), and *Lopesia* Rübsaamen (Lopesiini). The ingroups were represented for all the species within the genus *Bruggmanniella*, except for *Bruggmanniella byrsonimae* (Maia and Couri) due to its unknown larval stage. The list of species examined for the analysis can be found in the S1 Table. Most of the studied material was already preserved on mounted slides and were borrowed from the following institutions: Museu Nacional of Rio de Janeiro, Rio de Janeiro, Brazil (MNRJ), Museu de Zoologia of the Universidade de São Paulo, São Paulo, Brazil (MZSP), and National Museum of Natural History, Washington, DC, USA (NMNH). Literature data and photographs of the types unavailable for examination were used to aid in the character coding states. These photographs were received from the Collection of the Entomological Laboratory of the Kyushu University, Japan (KUEC).

### Character sampling

Morphological data of third-instar larva, pupa and adults were coded in a matrix. Our sampling resulted in 55 discrete characters and two continuous characters, totaling 57 characters. Of these, seven characters were coded from larvae, 17 from pupae (two continuous and 15 discrete), 33 from adults (25 of sex-specific characters) and one from galls. Continuous characters were used as it was not possible to categorize certain numeric features, nor to establish a range of values, avoiding arbitrary coding. These characters were treated according to unity-based normalization methods outlined in Thiele [32] and Goloboff et al. [33] (normalized data can be found in S2 Table). Nonapplicable and unknown data were entered in the matrix as ‘–’ and ‘?’, respectively (S3 Table). See S1 File for input matrix script.

### Phylogenetic analysis

The cladistic analysis was performed under the parsimony criterion with implicit weighting in TNT v1.1 (Willi Hennig Society Edition) [34]. Equal and implied weighting were implemented, the latter with values of *k* ranging from 3 to 20. All characters were treated as unordered. Tree searches were conducted using ‘Traditional Search’ with 1,500 replicas of Random Addition Sequence (RAS) combined with the branch-swapping: Tree Bisection and Reconnection (TBR) algorithm, saving 50 trees per replica. These values were chosen to reach the number of hits greater than 50 and to avoid overflow, so the most parsimonious trees could be chosen.

Relative Bremer support was calculated in TNT, retaining suboptimal trees by 10 steps, under implied weighting [35]. The suboptimal trees were obtained from a ‘traditional search’, using the ‘trees from RAM’ setting. The numbers of relative support are given by TNT in percentage, ranging from 0 to 100%. The performance of the characters was verified by the consistency index (CI) [36] and retention index (RI) [37]. Cladograms were rooted with *Lopesia andirae* Garcia, Lima, Calado and Urso-Guimarães (outgroup). The resulting tree was displayed in Adobe Illustrator CC software (17.0).

### Nomenclatural acts

The electronic edition of this article conforms to the requirements of the amended International Code of Zoological Nomenclature, and hence the new names contained herein are available under that Code from the electronic edition of this article. This published work and the nomenclatural acts it contains have been registered in ZooBank, the online registration system for the ICZN. The ZooBank LSIDs (Life Science Identifiers) can be resolved and the associated information viewed through any standard web browser by appending the LSID to the prefix "http://zoobank.org/". The LSID for this publication is: urn:lsid:zoobank.org:pub:90AF24F9-AEAD-458D-99DB-FDA1B12F9642. The electronic edition of this work was published in a journal with an ISSN, and has been archived and is available from the following digital repositories: PubMed Central, LOCKSS.

## Character descriptions

### Continuous characters

**0.** Length of pupal antennal horn (*ci* = 0.31; *ri* = 0.48; *fit* = 0.73). Pupal antennal horn length is measured from the base of the antennal sheath to the tip of the apical horn. In *Bruggmanniella* the length ranges from 0.13 mm (*B*. o*blita* Tavares) to 0.6 mm (*B*. *duguetiae* Urso-Guimarães and Amorim).

**1.** Length of pupal cephalic setae (*ci* = 0.30; *ri* = 0.33; *fit* = 0.72). The length of pupal cephalic setae is variable within *Bruggmanniella* ranging from 0 (*B*. *actinodaphnes* Tokuda and Yukawa) to 0.09 mm (*B*. *braziliensis* Tavares).

### Discrete characters

#### Larva

**2.** Prothoracic spatula; (0) present, (1) absent (*ci* = 1; *ri* = 1; *fit* = 1). The presence of prothoracic spatula is a synapomorphy to the family Cecidomyiidae. However, the absent state is only observed in the outgroup species *Bruggmannia acaudata* Maia.

**3.** Number of teeth of prothoracic spatula; (0) two, (1) three, (2) four (*ci* = 0.33; *ri* = 0.43; *fit* = 0.6). In *Bruggmanniella*, the number of teeth of prothoracic spatula is usually four, however, *B*. *actinodaphnes* and *B*. *cinnamomi* Tokuda and Yukawa have two teeth. *Bruggmanniella bumeliae* (Felt), *B*. *oblita* and the outgroup species *Illiciomyia yukawai* Tokuda are the only ones with three teeth.

**4.** Relative size of apical teeth; (0) same size, (1) outer larger than inner, (2) inner larger than outer (*ci* = 0.33; *ri* = 0.5; *fit* = 0.6). *Bruggmanniella* species are known for having prothoracic spatula with inner teeth larger than the outer ones. However, *B*. *brevipes* presents a spatula with the outer tooth larger than the inner ones. *Bruggmanniella actinodaphnes* and *B*. *cinnamomi* have equally sized teeth.

**5.** Lateral projection of stalk; (0) absent, (1) present (*ci* = 0.2; *ri* = 0.43; *fit* = 0.6). The stalk of prothoracic spatula usually has a lateral projection in Asphondyliini. Within *Bruggmanniella*, all species, except *B*. *maytenuse* (Maia and Couri), have this character.

**6.** Number of outer lateral papillae; (0) three, (1) two, (2) one (*ci* = 0.5; *ri* = 0.77; *fit* = 0.75). The number of outer lateral papillae in *Bruggmanniella* ranges from one to three. *Bruggamanniella brevipes* is the only species of the genus with three papillae. *Bruggmanniella actinodaphnes* and *B*. *cinnamomi* have two papillae. The other species have only one papilla.

**7.** Number of inner lateral papillae; (0) three, (1) two, (2) one (*ci* = 0.67; *ri* = 0.8; *fit* = 0.85). All *Bruggmanniella* species have one inner lateral papilla.

**8.** Papillae of terminal segment; (0) present; (1) absent (*ci* = 0.2; *ri* = 0.5; *fit* = 0.6). The state of character (0) is plesiomorphic to *B*. *braziliensis*, *B*. *bumeliae*, *B*. *ingae* Urso-Guimarães & Amorim and *B*. *maytenuse*. The state (1) is apomorphic to the other species of *Bruggmanniella*.

#### Pupa

**9.** Ratio of the width of the base by the height of the antennal horn; (0) width of base less than height, (1) same ratio (*ci* = 0.25; *ri* = 0; *fit* = 0.67). The width of the base and the height of the antennal horn are usually the same for all *Bruggmanniella* except for *B*. *oblita*.

**10.** Projection at the base of antennal horn; (0) absent, (1) present (*ci* = 1; *ri* = 1; *fit* = 1). The lateral projection at the base of antennal horn is present only in *B*. *cinnamomi* and *B*. *bumeliae*.

**11.** Outer edge of antennal horn; (0) smooth, (1) serrated, (2) toothed (*ci* = 0.33; *ri* = 0.67; *fit* = 0.6). The smooth outer edge is only observed in *B*. *cinnamomi*. The species *B*. *actinodaphnes*, *B*. *brevipes*, *B*. *bumeliae* and *B*. *ingae* have toothed outer edges. The others have serrated outer edges. States (1) and (2) may be linked to the type of tissue that the pupae need to open to emerge. The more rigid the plant tissue, the more serrated/toothed the horn is. Although *B*. *cinnamomi* has smooth outer edges and induces stem galls, the antennal horns are the largest one in length among *Bruggmanniella* species.

**12.** Inner edge of antennal horn; (0) smooth, (1) serrated (*ci* = 0.5; *ri* = 0.67; *fit* = 0.86). The inner edge is normally smooth in *Bruggmanniella* species. Two exceptions are *B*. *duguetia* and *B*. *doliocarpi* Maia.

**13.** Upper cephalic margins thickened; (0) present; (1) absent (*ci* = 0.25; *ri* = 0.67; *fit* = 0.67). In most species of *Bruggmanniella* the thickening of the upper cephalic margin is absent. This thickening, however, is present in *B*. *actinodaphnes*, *B*. *brevipes*, *B*. *cinnmamomi* and *B*. *doliocarpi*.

**14.** Frontal horn; (0) absent, (1) present (*ci* = 1; *ri* = 1; *fit* = 1). The frontal horn is only present in the outgroup species of *Asphondylia*.

**15.** Lateral facial papillae; (0) present; (1) absent (*ci* = 0.33; *ri* = 0; *fit* = 0.75). With a few exceptions (*B*. *cinnamomi* and *B*. *perseae* Gagné), the presence of lateral facial papillae is consistent within the genus.

**16.** Lower facial papillae; (0) present; (1) absent (*ci* = 0.5; *ri* = 0; *fit* = 0.86). With the exception of *B*. *cinnamomi*, all species of *Bruggmanniella* have lower facial papillae.

**17.** Dorsal papillae on the abdominal segments; (0) absent, (1) present (*ci* = 0.5; *ri* = 0; *fit* = 0.86). Only the outgroup species *Lopesia andirae* and *Parazalepidota clusiae* Maia do not have dorsal papillae on the abdominal segments.

**18.** Shape of abdominal spiracles; (0) rounded, (1) spiniform (*ci* = 0.5; *ri* = 0.87; *fit* = 0.86). The spiniform shape of abdominal spiracles is consistent within *Bruggmanniella* but not *B*. *ingae*.

**19.** Dorsal spines on second through sixth terminal tergites; (0) absent, (1) present (*ci* = 0.5; *ri* = 0; *fit* = 0.86). Dorsal spines on the abdominal segments are only absent in the outgroup species *L*. *andirae* and *B*. *acaudata*.

**20.** Dorsal spines on the median region of the eighth segment; (0) absent, (1) present (*ci* = 0.5; *ri* = 0.5; *fit* = 0.86). Within *Bruggmanniella* the presence of dorsal spines on the median region of the eighth segment is usual with the exception of *B*. *braziliensis*, *B*. *oblita*, and the outgroup *L*. *andirae*.

**21.** Arrangement of spines of the seventh segment; (0) irregular row, (1) regular row (*ci* = 0.25; *ri* = 0.5; *fit* = 0.67). *Bruggmanniella bumeliae* and *B*. *duguetiae* have an irregular arrangement of spines of the seventh segment. In the other species of the genus, the arrangement is regular.

**22.** Dorsal spines on the distal margin of the eighth segment; (0) absent, (1) present (*ci* = 0.5; *ri* = 0.75; *fit* = 0.86). Dorsal spines on the distal margin of the eighth segment are present in the outgroup species of *Asphondylia* and *B*. *acaudata*.

**23.** Terminal projections on the eighth abdominal segment; (0) absent, (1) present (*ci* = 0.5; *ri* = 0.67; *fit* = 0.86). Terminal projections on the eighth abdominal segment are normally absent within *Bruggmanniella*. However, these projections are present in *B*. *doliocarpi*, *B*. *duguetiae* and the outgroup *A*. *peploniae* Maia.

#### Adults

**24.** Flagellomeres; (0) long neck, (1) short neck (*ci* = 1; *ri* = 1; *fit* = 1). Flagellomeres with short neck are plesiomorphic to *L*. *andirae*. Long neck flagellomeres are apomorphic to all other species analyzed here.

**25.** Constriction at the base of flagellomeres; (0) absent, (1) present (*ci* = 1; *ri* = 1; *fit* = 1). A constriction at the base of flagellomeres is present only in the outgroup *I*. *yukawai*.

**26.** Shape of male flagellomeres; (0) binodal, (1) cylindrical (*ci* = 1; *ri* = 1; *fit* = 1). The binodal male flagellomere is a state only present in *L*. *andirae*.

**27.** Circumfila of male flagellomere; (0) with loops, (1) appressed (*ci* = 1; *ri* = 1; *fit* = 1). Only the outgroup species *L*. *andirae* has the circumfila of male flagellomere with loops.

**28.** First and second male flagellomeres; (0) separated, (1) fused (*ci* = 0.5; *ri* = 0; *fit* = 0.86). First and second male flagellomeres are observed in *B*. *acaudata* and *P*. *neolitseae*.

**29.** Length of male flagellomeres; (0) equal size, (1) shortened distally (*ci* = 0.25; *ri* = 0.4; *fit* = 0.67). Male flagellomeres shortened distally are plesiomorphic to all *Bruggmanniella* species. Equally sized flagellomeres are apomorphic to *Pseudasphondylia* species except in *P*. *kiritanii* Tokuda and Yukawa.

**30.** Circumfila of female flagellomere; (0) not sinuous, (1) sinuous (*ci* = 0.2; *ri* = 0.55; *fit* = 0.6). Within *Bruggmanniella*, the sinuous circumfila of female flagellomere is only found in *B*. *actinodaphnes*, *B*. *brevipes* and *B*. *cinnamomi*.

**31.** First and second female flagellomeres; (0) separated, (1) fused (*ci* = 0.2; *ri* = 0.33; *fit* = 0.6). Separated first and second female flagellomeres are plesiomorphic to all species of *Bruggmanniella*. The fused state is only observed in *B*.*acaudata*, *P*. *clusiae* and *Pseudasphondylia* species with the exception of *P*. *rauwolfiae* Coutin.

**32.** Length of female flagellomeres; (0) equal size, (1) shortened distally (9th– 12^th^) (*ci* = 1; *ri* = 1; *fit* = 1). Equally sized flagellomeres are plesiomorphic to *L*. *andirae*. Flagellomeres 9th– 12^th^ shortened distally are apomorphic to all other species analyzed here.

**33.** Number of palpal segments; (0) four, (1) three, (2) two, (3) one (*ci* = 0.33; *ri* = 0.25; *fit* = 0.5). The number of palpal segments comprises all segments with setae, from the basal one, which is regarded as a palpiger in some descriptions, to the last segment (see Tokuda and Yukawa, 2005). The number of palpomeres is usually three within *Bruggmanniella* genus, with a few exceptions *B*. *actinodaphnes*, *B*. *duguetiae* and *B*. *miconia* **sp. nov.**

**34.** Apical spur; (0) present, (1) absent (*ci* = 0.5; *ri* = 0; *fit* = 0.86). The presence of apical spur on the first tarsomere is plesiomorphic to *Bruggmanniella*. Only *Schizomyia macrocapillata* Maia and *I*. *yukawai* have an apical spur.

**35.** Tarsal claw; (0) toothed, (1) not toothed (*ci* = 1; *ri* = 1; *fit* = 1). The toothed tarsal claw is a sole state to *L*. *andirae*.

**36.** Length of empodium; (0) less than claw, (1) same length, (2) larger than claw (*ci* = 0.2; *ri* = 0.35; *fit* = 0.4). The length of empodium is variable within *Bruggmanniella*. State (0) is found in *B*. *oblita* and *B*. *miconia* **sp. nov.** State (2) is found in *B*. *braziliensis* and *B*. *cinnamomi*. State (1) is found in other species.

**37.** Pulvilli; (0) absent, (1) present (*ci* = 0.25; *ri* = 0.4; *fit* = 0.67). Pulvilli is present in *B*. *actinodaphnes*, *B*. *braziliensis*, *B*. *cinnamomi*, *B*. *oblita*, and *B*. *perseae*.

**38.** Parameres; (0) absent, (1) present (*ci* = 0.33; *ri* = 0.8; *fit* = 0.75). Parameres are only present in the Oriental/Palearctic species of *Bruggmanniella*. State (1) is also found in *I*. *yukawai* and all *Pseudasphondylia* species. Tavares (1909) does not mention whether or not *B*. *braziliensis* has parameres, therefore we coded as “?”. Males of *B*. *duguetiae* and *B*. *oblita* are unknown.

**39.** Mesobasal lobe on gonocoxite; (0) present, (1) absent (*ci* = 1; *ri* = 1; *fit* = 1). The mesobasal lobe is only present in the outgroup species *L*. *andirae*.

**40.** Aedeagus; (0) wide, (1) narrow (*ci* = 0.5; *ri* = 0; *fit* = 0.86). State (0) is found in the outgroup species *L*. *andirae* and *B*.*acaudata*.

**41.** Apex of gonocoxite; (0) not exceeding the insertion of gonostylus, (1) exceeding the insertion of gonostylus (*ci* = 0.25; *ri* = 0.7; *fit* = 0.67). In *Bruggmanniella*, the apex of gonocoxite exceeds the insertion of gonostylus in *B*. *actinodaphnes* and *B*. *cinnamomi*.

**42.** Gonostylus; (0) clavate, (1) rounded, (2) rectangular (*ci* = 0.67; *ri* = 0.67; *fit* = 0.86). All species of *Bruggmanniella* have rounded gonostylus except for *B*. *brevipes*, which has rectangular gonostylus.

**43.** Teeth of gonostylus; (0) present, (1) absent (*ci* = 1; *ri* = 1; *fit* = 1). All species of *Bruggmanniella* have teeth on the gonostylus. The absence of teeth is a state found in the outgroup species of Schizomyiina, *B*. *acaudata* and *S*. *macrocapillata*. Schizomyiina species have gonostylus with comb-like spines arranged at the apex, not a single tooth.

**44.** Teeth of gonostylus; (0) unified, (1) separated (*ci* = 1; *ri* = 1; *fit* = 1). State (1) is present in all *Bruggmanniella* species. This is also found in the outgroup *I*. *yukawai* and *Pseudasphondylia*.

**45.** Spines of gosnostylus; (0) absent, (1) present (*ci* = 0.5; *ri* = 0.5; *fit* = 0.86). Spines on the gonostylus are found in the outgroup species *B*. *acaudata*, *S*. *macrocapillata* and *P*. *clusiae*. The spines present in *P*. *clusiae* are separated and arranged at the apex of the gonostylus.

**46.** Cerci; (0) unified, (1) separated (*ci* = 0.33; *ri* = 0.5; *fit* = 0.75). The cercus is normally unified in *Bruggmanniella* with a few exceptions, *B*. *actinodaphnes* and *B*. *cinnamomi*.

**47.** Shape of cerci; (0) triangular, (1) rounded, (2) rectangular, (3) reniform (*ci* = 0.5; *ri* = 0.25; *fit* = 0.67). All species of *Bruggmanniella* have rounded cerci except for *B*. *cinnamomi*, which has triangular cerci. States (2) and (3) are found among the outgroup.

**48.** Hypoproct; (0) bilobed, (1) unilobed (*ci* = 1; *ri* = 1; *fit* = 1). Unilobed hypoproct is apomorphic to *B*. *braziliensis*, *B*. *ingae* and *B*. *maytenuse*.

**49.** Shape of hypoproct; (0) rounded, (1) triangular, (2) rectangular (*ci* = 0.67; *ri* = 0; *fit* = 0.86). The majority of *Bruggmanniella* species has rounded hypoproct but *B*. *maytenuse* is rectangular.

**50.** Ratio of cerci and hypoproct; (0) cerci shorter than hypoproct, (1) cerci larger than hypoproct, (2) same size (*ci* = 0.33; *ri* = 0.5; *fit* = 0.6). The length of the cerci in relation to the hypoproct varies among *Bruggmanniella* species. State (0) is found in *B*. *brevipes* and *B*. *miconia* **sp. nov.** State (2) is only found in *B*. *maytenuse*. The other species have cerci larger than hypoproct.

**51.** Ovipositor; (0) not protrusible (1) protrusible (*ci* = 1; *ri* = 1; *fit* = 1). Protrusible ovipositor is plesiomorphic to *L*. *andirae*. Not protrusible ovipositor is apomorphic to all other species analyzed here.

**52.** Ovipositor; (0) ovoid, (1) tubular, (2) piriform (*ci* = 0.67; *ri* = 0; *fit* = 0.86). The tubular ovipositor is consistent within *Bruggmanniella*, however, *B*. *braziliensis* and *B*. *duguetiae* have a piriform ovipositor.

**53.** Length of ovipositor compared to seventh sternite; (0) same length, (1) larger (*ci* = 1; *ri* = 1; *fit* = 1). Only the outgroup species *L*. *andirae* has ovipositor and seventh sternite of equal size.

**54.** Cerciform lobe at distal margin of eighth abdominal segment; (0) absent, (1) present (*ci* = 1; *ri* = 1; *fit* = 1). State (0) is only found in the outgroup species *L*. *andirae*, *B*. *acaudata*, and *S*. *macrocapillata*.

**55.** Apical region of ovipositor; (0) membranous, (1) sclerotized (*ci* = 1; *ri* = 1; *fit* = 1). The sclerotized apical region of the ovipositor is a synapomorphy to subtribe Asphondyliina. State (0) is only observed in the outgroup species *L*. *andirae*, *B*. *acaudata*, and *S*. *macrocapillata*.

**56.** Gall; (0) leaf, (1) stem, (2) flower bud, (3) fruit (*ci* = 0.3; *ri* = 0.56; *fit* = 0.46). Galls on stems are consistent within *Bruggmanniella*. *Bruggmanniella brevipes* is the only species inducing galls on leaf buds. *B*. *ingae* induces galls on flower bud while *B*. *maytenuse* and *B*. *perseae* induce galls on fruits.

## Results and discussion

### Phylogenetic analysis

**Fig 1:**
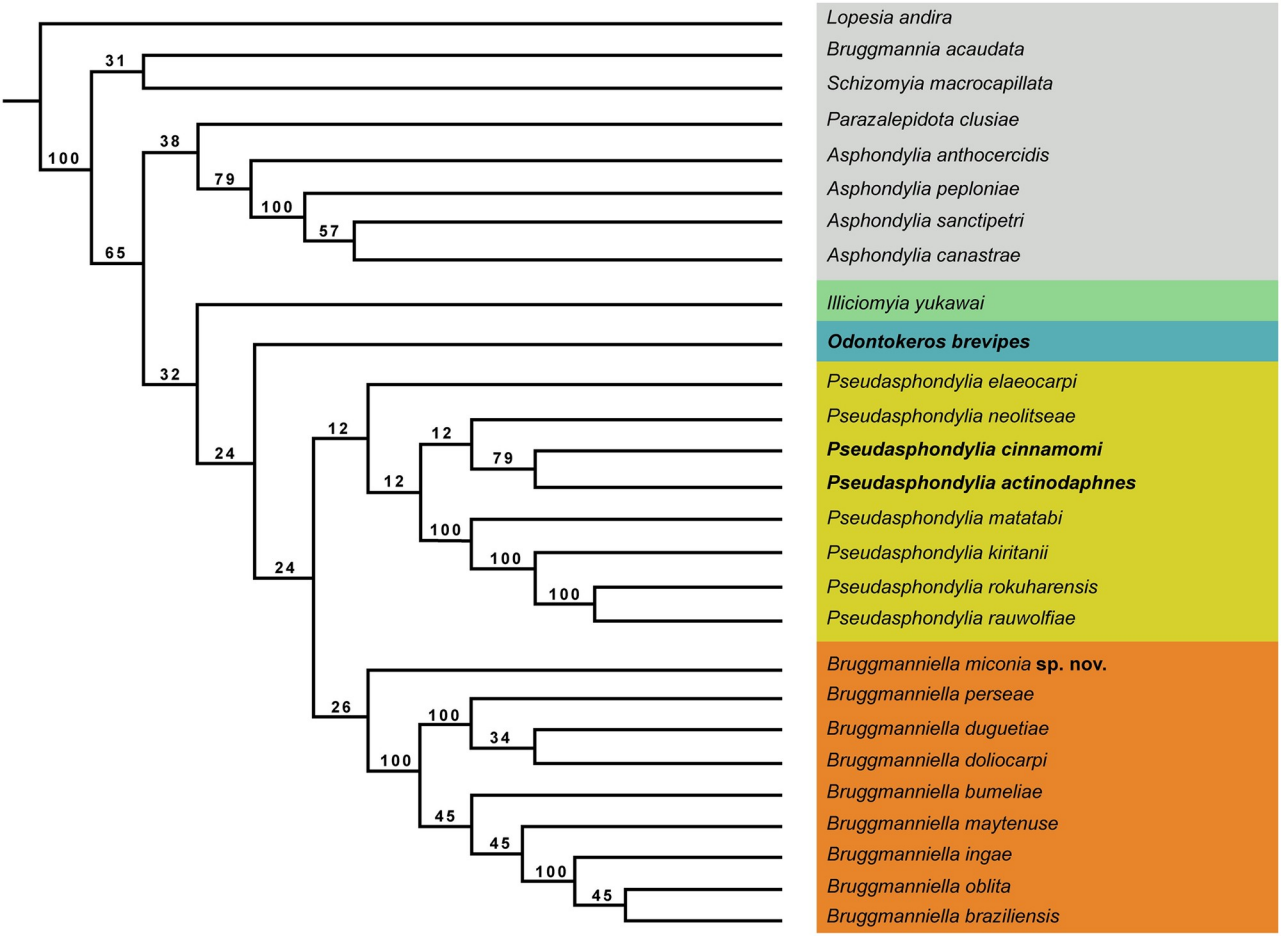
Phylogenetic hypothesis for *Bruggmanniella* Tavares, 1909. Result from parsimony analysis of the morphological characters under implied weight (k = 6); Relative Bremer support values (in percentage) on branches.

The phylogenetic analysis under equal and implied weights produced a single parsimonious tree (S1 and S2 Figs), with both analyses showing *Bruggmanniella* as the sister group of *Pseudasphondylia*. The tree found for *k* = 6–20 seems to be stable with the resulting topology for the ingroup taxa with no changes in *k* values. The MPT for *k* = 6 (fit = 35.91, length = 175 steps, CI = 0.41, RI = 0.56) is shown in Fig 1.

In this study, three synapomorphies support the monophyly of the genus *Bruggmanniella*: (i) thickening absent in the pupal upper cephalic margin, (ii) circumfila of female flagellomeres not sinuous, and (iii) parameres absent.

Our results show two main groups within the genus. The first with *B*. *perseae*, *B*. *duguetiae* and *B*. *doliocarpi*. The character supporting this clade is the length of the pupal horn, which varies in a morphocline from 0.4 to 0.6 mm. The second clade that comprises *B*. *bumeliae*, *B*. *maytenuse*, *B*. *ingae*, *B*. *oblita*, and *B*. *braziliensis* is supported by the absence of terminal papillae in the third larval instar. This analysis also recovers *B*. *miconia* **sp. nov.** as the sister group of all other species of *Bruggmanniella* in the basal clade of the genus. The analysis did not recover *B*. *actinodaphnes*, *B*. *cinnamomi*, and *B*. *brevipes* in the *Bruggmanniella* genus.

Tokuda and Yukawa [21, 22] state that the similarity between *Bruggmanniella* and *Pseudasphondylia* is based on the presence of the two separated teeth in the gonostylus and in the presence of parameres. One of the synapomorphies of *Bruggmanniella* resultant of this analysis is the absence of parameres, which is present in all Schizomyiina species, but missing in the most genera of Asphondyliina. They are present in all species of *Pseudasphondylia* and absent in all Neotropical species of *Bruggmanniella*. The Oriental/Palearctic species of *Bruggmanniella–B*. *actinodaphnes* and *B*. *cinnamomi*–have parameres, and in our results, they are placed among the species of *Pseudasphondylia*. They form a sister clade with *P*. *neolitseae*, based on the homoplastic synapomorphies: (i) prothoracic larval spatula with two teeth of the same size, (ii) spatula with two outer lateral papillae, and (iii) cerci larger than hypoproct. With the inclusion of *B*. *actinodaphnes* and *B*. *cinnamomi* in *Pseudasphondylia*, the monophyly of *Bruggmanniella* is recovered and supported by the following synapomorphies: (i) first and second female flagellomeres fused and (ii) male flagellomeres gradually shortened. The results of our analysis support the transference of *B*. *actinodaphnes* and *B*. *cinnamomi* to *Pseudasphondylia* Monzen, named *Pseudasphondylia actinodaphnes* (Tokuda and Yukawa, 2006) **new comb.** and *Pseudasphondylia cinnamomi* (Tokuda and Yukawa, 2006) **new comb.**

*Bruggmanniella brevipes* Lin, Yang and Tokuda appears in our results in the base of (*Bruggmanniella* + *Pseudasphondylia*). *Bruggmanniella brevipes* shares with *Pseudasphondylia* and the other *Bruggmanniella* species, the two separated teeth of the gonostylus in the male and the absence of frontal horns on pupa. However, *B*. *brevipes* differs by having prothoracic larval spatula with four teeth with the outer teeth larger than the inner ones. Also, *B*. *brevipes* has shorter legs than its congeners and induces bud galls on *Neolitsea parvigemma* (Lauraceae), which is endemic in Taiwan.

The results of this study indicate that *B*. *brevipes* does not belong to any described genus of Asphondyliina, so as a consequence of this analysis we propose the erection of a new genus named *Odontokeros* Garcia, Lamas and Urso-Guimarães **gen. nov.**, to which this species will be transferred based on the following synapomorphies: presence of prothoracic spatula with four teeth in larva, with the outer teeth larger than the inner ones, and shorter legs in adults. *Odontokeros brevipes* (Lin, Yang and Tokuda) **comb. nov.** is a monotypic genus with *Bruggmanniella brevipes* as the type species by present designation.

*Illiciomyia* Tokuda is recovered as a monotypic genus, as the sister group of the clade formed by (*Odontokeros* **gen. nov.** + (*Bruggmanniella* + *Pseudasphondylia*)). Moreover, these four genera share the two separated teeth of the gonostylus and the absence of frontal horns on pupa. Tokuda [25] argued that *Illiciomyia yukawai* is phylogenetically diverging from *Bruggmanniella* and *Pseudasphondylia* based on the shallow constriction of male flagellomeres, less appressed male circumfila, absence of apical spur on the first tarsomere of all legs, and larva with five lateral papillae. Our analysis supports these arguments and additionally points out the difference in the consistency and presence/absence of parameres, which are absent in *Bruggmanniella*, membranous in *Pseudasphondylia* and in *Odontokeros* **gen. nov.**, and sclerotized in *Illiciomyia*.

#### Remarks on geographical distribution and gall maker-host plant association

**Fig 2:**
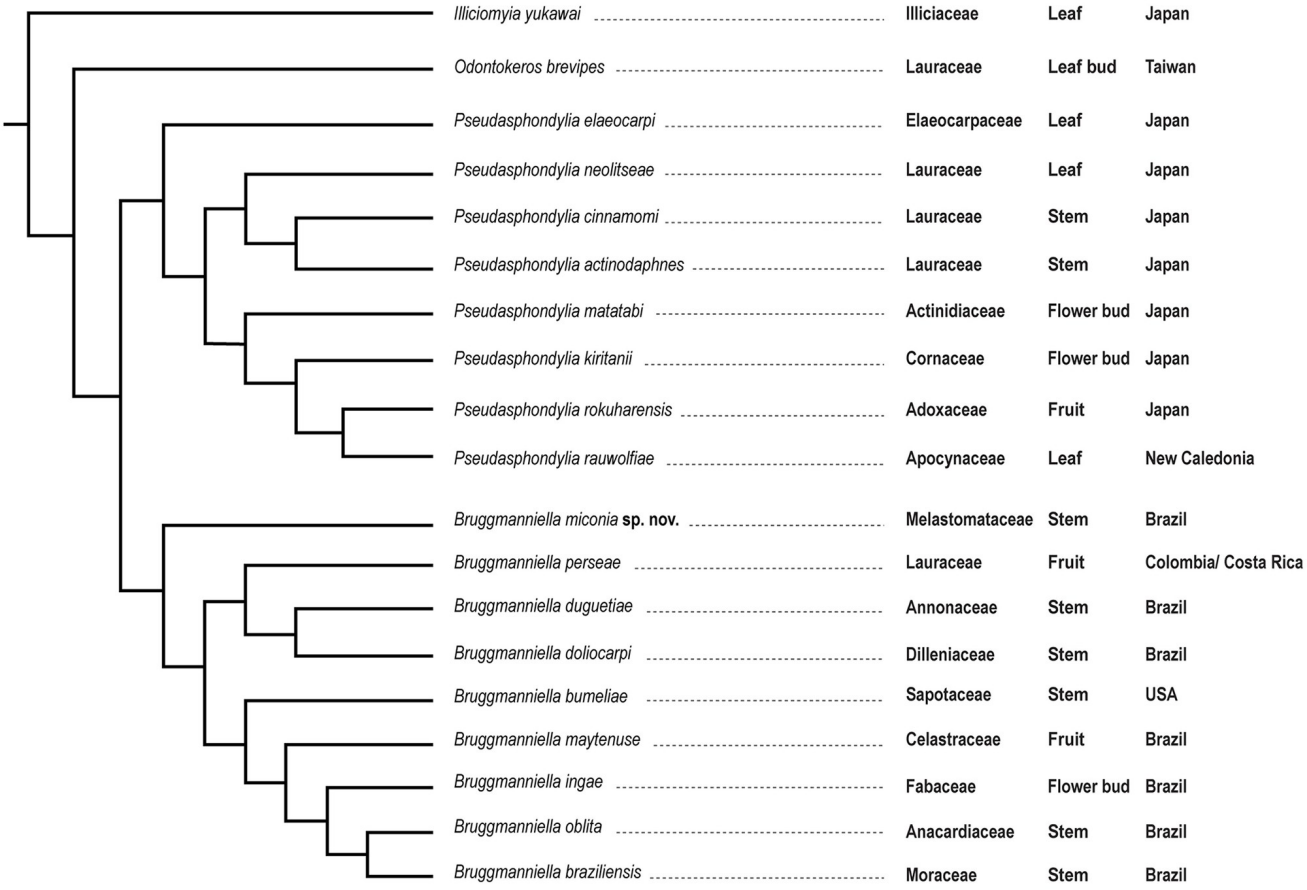
Phylogenetic hypothesis for *Bruggmanniella* Tavares, 1909 with their host families, galls and area of occurrence.

The result of this cladistic analysis allows us to discuss some aspects of the gall inducer-host plant association of *Bruggmanniella*, *Pseudasphondylia*, *Illiciomyia*, and *Odontokeros* **gen. nov.** Information on host plant families and geographical location data for each species were added to the resulting topology (Fig 2). The genus *Pseudasphondylia* has its distribution restricted to the Australian, Oriental, and Palearctic regions, while *Bruggmanniella* is now restricted to the New World, mostly to the Neotropics. Most *Bruggmanniella* species have their distribution concentrated in the South-Eastern Region of Brazil. The same is true to *Pseudaphondylia*, *Illiciomyia*, and *Odontokeros* **gen. nov.** in which most species are recorded in Japan. This may reflect the lack of sampling in other areas/regions, as well as the concentration of experts, and consequently of collection efforts, in these specific regions.

Galls on stems are much more frequent in *Bruggmanniella* (60%) than in its sister group *Pseudasphondylia* (25%—including *B*. *actinodaphines* and *B*. *cinnamomi*). Fifty percent of *Pseudasphondylia* species, the monotypic genera *Illiciomyia* and *Odontokerus* **gen. nov.** induce galls on leaves. The induction on stems appears as a homoplastic synapomorphy to the genus *Bruggmanniella*. In addition, the clade *P*. *neolitseae* + (*P*. *actinodaphnes* + *P*. *cinnamomi*) induces galls on species of the same family of the host plant, Lauraceae, in the geographic distribution in Japan, giving consistency to the proposed hypothesis.

Tokuda and Yukawa [22] also argued that *B*. *perseae* seems to be morphologically close to the congeners of the Neotropical Region than those of the Oriental and Palearctic regions, due to the presence of prothoracic larval spatula with four teeth and male cerci basally fused. Our analysis corroborates this perception. *Bruggmanniella perseae* is positioned among the Neotropical species, which reinforces the hypothesis that the induction of galls on Lauraceae arose independently in the Neotropical and Oriental/Palearctic regions.

Our results do not show patterns of coevolution or co-speciation among gall inducers and families of host plants in New World species. Each of the ten *Bruggmanniella* species induces galls on different families of plants. Möhn [38] had already stated that there was no “parallelism” between the evolution of “Asphondyliidi” and their host plants. Also, the morphocline of growing development of the antennal horn in pupae indicates a preference of inducing gall on stems instead of leaves, most commonly in Oriental and Eastern Palearctic *Pseudasphondylia* species.

Hypotheses about when and where these species initially diverged are difficult to state. Although most Asphondyliini species are distributed throughout the Neotropical Region, there is no information about when the association of these species with their host plants has begun. Biological data on life cycle strategies, behavior, host interactions and physiology for *Bruggmanniella* species are still unknown or incomplete. More complex analyses on biogeography and co-speciation could offer a better understanding of these issues.

### Taxonomy

We present below the new taxonomic treatment for *B*. *actinodaphnes*, *B*. *cinnamomi* and *B*. *brevipes*; the description of the new species and the identification keys.

***Pseudasphondylia actinodaphnes* (Tokuda and Yukawa, 2006) comb. nov**.

*Bruggmanniella actinodaphnes* Tokuda and Yukawa, 2006: 633–635, Figs 2A, 2B, 3A and 3B.

**Fig 3:**
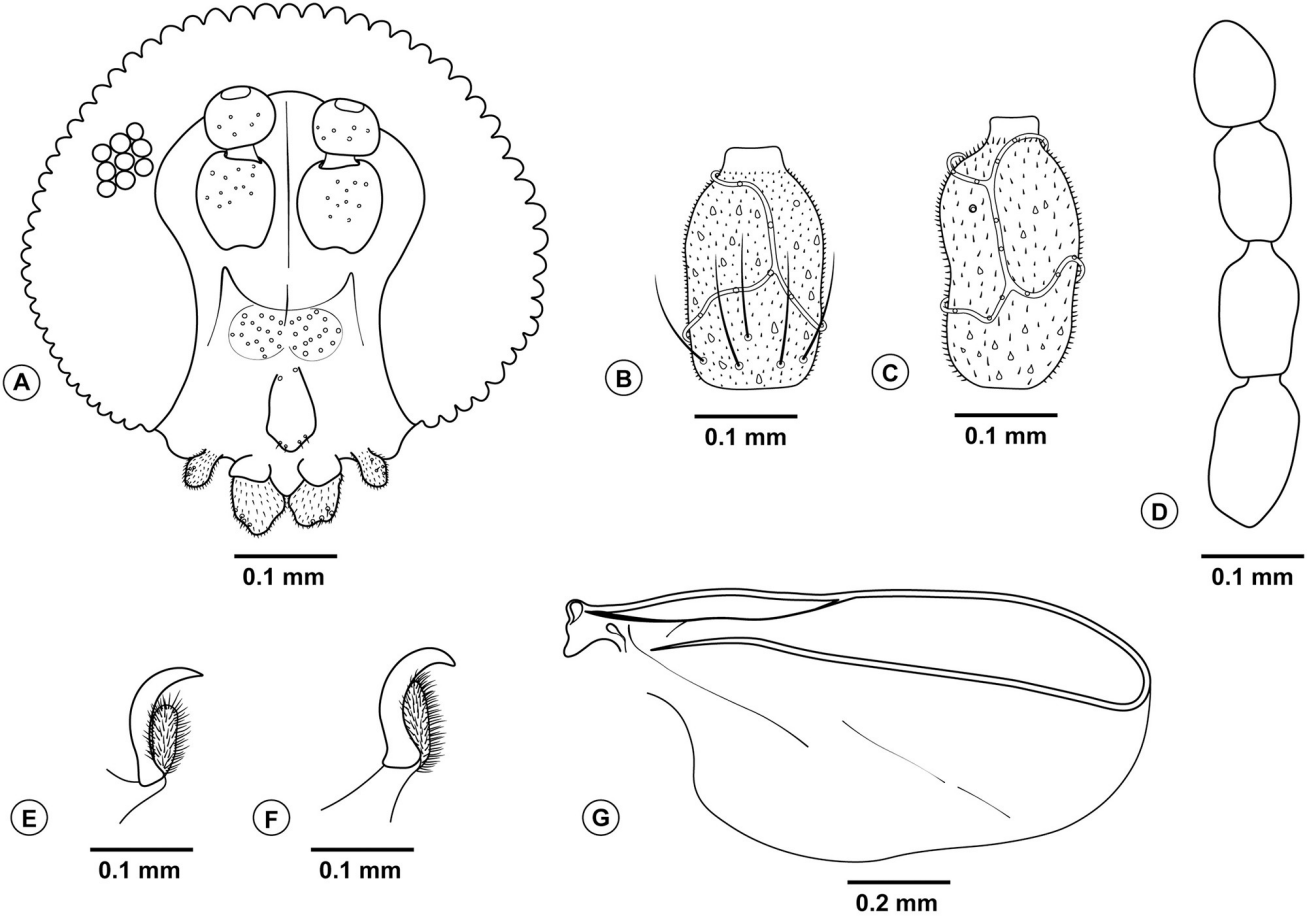
*Bruggmanniella miconia* sp. nov., male and female. **(A)** Male head (frontal view) **(B)** 5th female flagellomere. **(C)** 5th male flagellomere. **(D)** 9–12th female flagellomeres. **(E)** Male tarsal claw and empodium. **(F)** Female tarsal claw and empodium. **(G)** Female wing.

**Host Plant.** *Litsea coreana* (= *Actinodaphne lancifolia*) (Lauraceae).

**Distribution.** Honshu, Kyushu and Shikoku, Japan.

***Pseudasphondylia cinnamomi* (Tokuda and Yukawa, 2006) comb. nov**.

*Bruggmanniella cinnamomi* Tokuda and Yukawa, 2006: 630–633, Figs 2C, 3D, 3C and 3D.

**Host Plant.** *Cinnamomum japonicum* (Lauraceae).

**Distribution.** Southwest islands, Japan.

***Odontokeros*** Garcia, Lamas and Urso-Guimarães **gen. nov.**

urn:lsid:zoobank.org:act:CC869A4C-2D7B-4F09-80C0-F96861836D71

**Type species.** *Bruggmanniella brevipes* Lin, Yang and Tokuda, 2019: 205–206, Figs 1B–1E, 2A–2F, by original designation (type locality: Jinshuiying trail, Pingtung, Taiwan).

**Diagnosis.** Prothoracic spatula with 4- teeth, outer teeth larger than the inner ones; pupa with deeply toothed antennal horns and prothoracic spiracles well developed, upper and frontal horns absent, presence of thickening on pupal cephalic margin; antenna with 12 flagellomeres, male genitalia with two well-developed gonostylar teeth, hypoproct deeply bilobed, parameres present; cerci-like lobes, short female legs [2].

**Etymology.** The name *Odontokeros* is a reference to the pupal antennal horns, which have the outer edge deeply toothed (Gr. *Odonto*, tooth; *keros*, horn).

**Host plant.** *Neolitsea parvigemma* (Lauraceae).

**Distribution.** Southern, Taiwan.

**Genus *Bruggmanniella* Tavares, 1909.** Type species: *Bruggmanniella braziliensis* Tavares, 1909, by orig. des.

**Diagnosis.** Prothoracic larval spatula with 3 or 4 teeth, inner teeth (or tooth) larger than outer ones; pupa with antennal horns and well-developed prothoracic spiracles, absent upper and frontal horns, lack of thickening on pupal cephalic margin; male genitalia with separated gonostylar teeth, parameres absent; cerci-like lobes present on female abdominal segment VIII [39–41].

*Bruggmanniella miconia* Garcia, Lamas and Urso-Guimarães **sp. nov.**

urn:lsid:zoobank.org:act:80866883-69C9-46F2-8361-52F60D69A584

(Figs 3–6)

**Fig 4:**
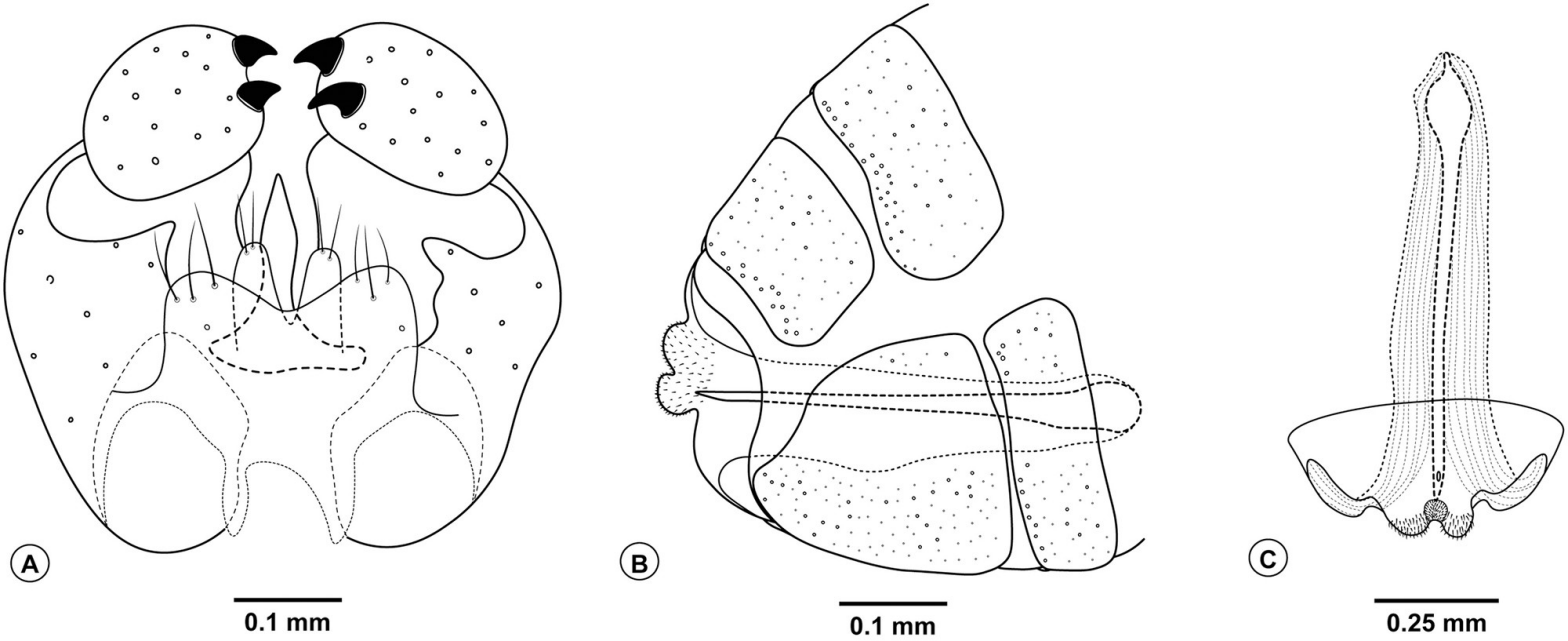
*Bruggmanniella miconia* sp. nov. **(A)** Male terminalia (dorsal view). **(B)** Ovipositor (lateral view). **(C)** Ovipositor (dorsal view).

**Fig 5:**
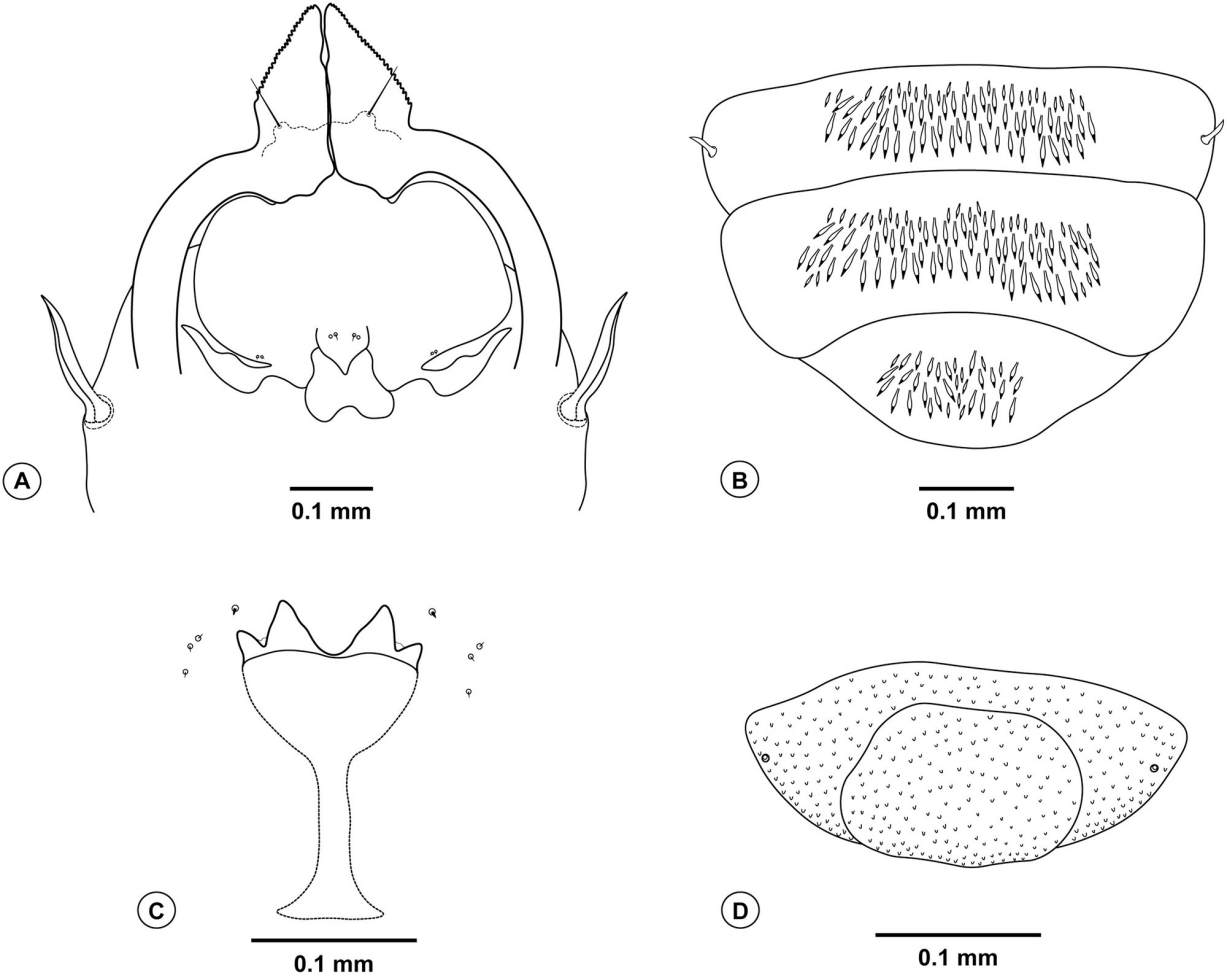
*Bruggmanniella miconia* sp. nov., pupa and larva. **(A)** Pupal head (ventral view). **(B)** 6–8th pupal abdominal segments (dorsal view). **(C)** Larval prothoracic spatula and lateral papillae (ventral view). **(D)** Larval terminal segment (dorsal view).

**Fig 6:**
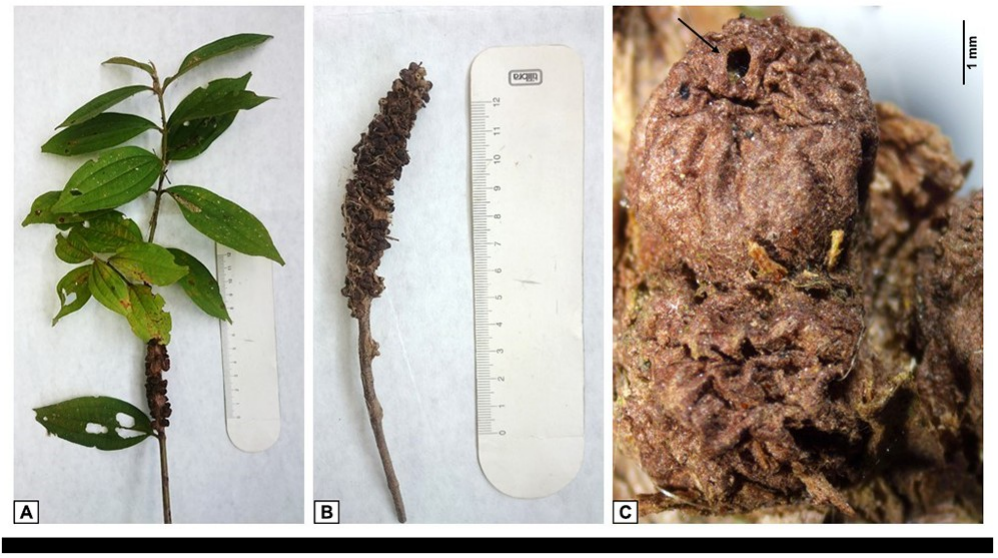
Host plant. **(A)** Branch of *Miconia* cf. *cinnamomifolia*. **(B)** Galls of *Bruggmanniella miconia* **sp. nov. (C)** Gall in detail showing the small hole positioned at the apex for the emergence of the adult. Scale bar: 1 mm.

**Diagnosis.** The new species can be easily segregated from the congeners by the following combination of characters: prothoracic spatula with well-spaced inner teeth, terminal segment with absence of papillae; right triangle-shaped antennal horns and edge minutely serrate, cephalic setae long, abdominal segments with conspicuous spines; flagellomeres short and rounded with two appressed circumfila, connected to one another, on both sexes; subcoastal vein convex; tarsal claws simple and longer than empodium; hypoproct deeply bilobed with apically rounded lobes, cercus slightly bilobed, twice as wide as hypoproct, aedeagus narrow at base and pointed at apex; needle-like ovipositor with cercilike lobes present.

**Description. Male (inside pupal exuvia).** *Head* (Fig 3A): Frons densely setose; clypeus with six setae; labellum with four setae. Palpus one-segmented. Antenna with 12 flagellomeres; scape and pedicel globoid and setose; flagellomeres with appressed circumfila (Fig 3B). *Thorax*: Legs with dense blackish scales; first tarsomere of all legs with apical spur; tarsal claw simple, similar in size in all legs; empodium shorter than claws and not reaching its curvature, pulvilli absent (Fig 3E). *Terminalia* (Fig 4A): gonocoxites large, gonostylus rounded with its length 0.6 times larger than the width, gonostylus teeth completely divided; parameres absent; cercus twice as wide as hypoproct, slightly bilobed, each lobe rounded apically and with three setae at apex; hypoproct rounded, larger than cercus, deeply bilobed, each lobe with two setae at apex; lanceolate aedeagus, narrow at base with pointed apex.

**Female.** Brown. Length: 1.8 mm (n = 1) (ovipositor not extruded). *Head*: Frons, clypeus, labellum, and palpus as in male. Antenna with 12 flagellomeres; scape and pedicel globoid and setose; flagellomeres with two appressed circumfila connected to each other (Fig 3C); flagellomeres 9–12 subequal in length (Fig 3D). *Thorax*: Scutum and scutellum dark brown; anepimeron setose; other pleura bare. Legs with dense blackish scales; first tarsomere of all legs with apical spur; tarsal claw simple, similar in size in all legs, empodium shorter than claw but reaching its curvature, pulvilli absent (Fig 3F). Wings length 2.1 mm and 1.08 mm in width. Venation as in Fig 3G, subcostal convex. *Abdomen* (Fig 4B): First through seventh tergites rectangular and sclerotized; First through sixth sternites rectangular and sclerotized, seventh sternite longer than the others and strongly striated; tergites and sternites without trichoid sensilla. *Ovipositor* (Fig 4B and 4C): protrusible, basal portion membranous and striated (0.7 mm length), apical portion sclerotized and aciculate (0.6 mm length); cercilike lobes present at eight abdominal tergites.

**Pupa** (Fig 5A and 5B). Integument light brown. Length: 2.4–2.8 mm (n = 10). Cephalic setae long (0.06 mm). Antennal horns elongate (0.3 mm length), well-sclerotized, right triangle-shaped, outer edges minutely serrate and inner edges smooth. Upper facial margin not thickened, two pairs of lower facial papillae (one setose and one bare), two pairs of lower lateral setose papillae. Prothoracic spiracles with 0.2 mm length. Leaflets reaching 1/3 of the fifth abdominal segment; all pairs of legs reaching the sixth abdominal segment. Abdominal tergites of the second through eighth segments with dorsal rows of conspicuous spines mesally positioned and different in size; four dorsal papillae with setae situated below the most posterior row of spines. Eighth tergite with fewer spines than the seventh tergite. Abdominal spiracles of the first, seventh and eighth segments sessile. Those of the second through sixth segments well-developed and spiniform.

**Larva** (Fig 5C and 5D). Yellowish. Integument delicate and hyaline, entirely covered by micro spicules. Length: 1.8–2.5 mm (n = 10). Prothoracic larval spatula (0.3 mm length) with four teeth, inner teeth well-spaced and longer than outer ones. Cervical and sternal papillae with short setae. Three lateral papillae present on each side of the spatula, all setose (one internal and two external). Ventral anus. Terminal segment with papillae not distinguishable from spicules.

**Galls and biology.** Globoid, glabrous; reddish when young and brown when mature. Induced on stems of *Miconia* cf. *cinnamomifolia* (Melastomataceae) (Fig 6A). The galls are unilocular and individualized, occurring in clusters ranging from 10 to more than 100 galls per branch (Fig 6B). The larva pupates in the gall and the adults emerge from a small hole positioned at the apex carved by the antennal horns of the pupa (Fig 6C). Non-identified pupa and female of *Schizomyia* Kieffer were also found in the galls of the new species. This is the first record of *Schizomyia* on Melastomataceae and as inquiline in *Bruggmanniella* galls.

**Etymology.** The specific name “*miconia*” is in apposition, referring to the name of the host plant genus.

**Type material. Holotype**–Female (n = 1). [Permanent slide] (MZSP). Brazil: São Paulo, Sorocaba (23°35'09.4" S; 47°30'59.9" W), 02.X.2015, on stems of *Miconia* cf. *cinnamomifolia* (Melastomataceae), Ansaloni LS col.; **Paratypes.** 10 males (inside pupal exuvia), 15 pupae, 10 larvae, same locality, col. in 08.i.2019, Garcia CA. col; 5 exuviae, Cordeiro A. col.

**DNA.** COI analysed for two pupae. GenBank accession number MN686345 (543 bp).

**Remarks.** *Bruggmanniella miconia* **sp. nov.** is configured as a new species belonging to the genus *Bruggmanniella* by the following characters: prothoracic larval spatula with four teeth, with inner teeth larger than the outer ones; pupal antennal horns strongly sclerotized and well-developed; gonostylus teeth completely divided, parameres absent, needle-like ovipositor with cercilike lobes present and subcoastal vein convex.

The new species differs from all its congeners by the unique shape of the male and female flagellomeres, which are shorter and rounded, with two appressed circumfila connected to one another. *Bruggmanniella miconia* **sp. nov.** also differs from *B*. *perseae*, B. *oblita* and *B*. *braziliensis* by the absence of pulvilli (present in these species) and the cerci shorter than hypoproct. *Bruggmanniella perseae* and *B*. *braziliensis* have cerci longer than hypoproct. The subcoastal vein of the new species is convex, resembling *Zalepidota piperis* Rübsaamen (Asphondyliini). *Bruggmanniella byrsonimae*, *B*. *maytenuse*, and *B*. *perseae* have a straight subcoastal vein and a concavity between Sc and R_1_. Both *B*. *duguetiae* and *B*. *ingae* have a slightly convexity on Sc, but they have CuA_1_ and CuA_2_ veins complete, while the wing of *B*. *miconia* **sp. nov.** has CuA_1_ and CuA_2_ veins incomplete.

The pupae of *B*. *miconia* **sp. nov.** is distinguishable from those of *B*. *perseae* and *B*. *duguetiae* by the antennal horn straight laterally except for serrations on the outer edge. *Bruggmanniella duguetiae* has an antennal horn which is concaved laterally, while *B*. *perseae* has a convex antennal horn. Also, *B*. *duguetiae* has projections present in the eighth abdominal segment, as well as *B*. *bysonimae*, which are absent in *B*. *miconia* **sp. nov.** *Bruggmanniella maytenuse* has a laterally straight antennal horn, but this species has the upper facial margin thickened while the pupa of *B*. *miconia* **sp. nov.** has no thickening on the upper facial margin.

The larvae of the new species and of *B*. *doliocarpi* shares the prothoracic spatula with well-spaced inner teeth, but *B*. *miconia* **sp. nov.** differs from *B*. *doliocarpi* by having inner and outer teeth with rounded apices, and a narrow long stalk. The terminal papillae are also absent on *B*. *miconia* **sp. nov.**, *B*. *oblita* and *B*. *perseae*. However, the prothoracic spatula of *B*. *oblita* has three teeth (the new species has four teeth) and *B*. *perseae* has inner and outer teeth of the same size.

#### Identification keys to the new world species of *Bruggmanniella*

The identification keys presented here are based on that of Gagné et al. [42]. Three keys are presented below, each for a different life stage, including all New World species of *Bruggmanniella*. Data on galls and distribution were also included in the adult key. It was not possible to key females of all species since they are very similar to each other.

### Identification key to the third instar of the known larvae of *Bruggmanniella*

**Fig 7:**
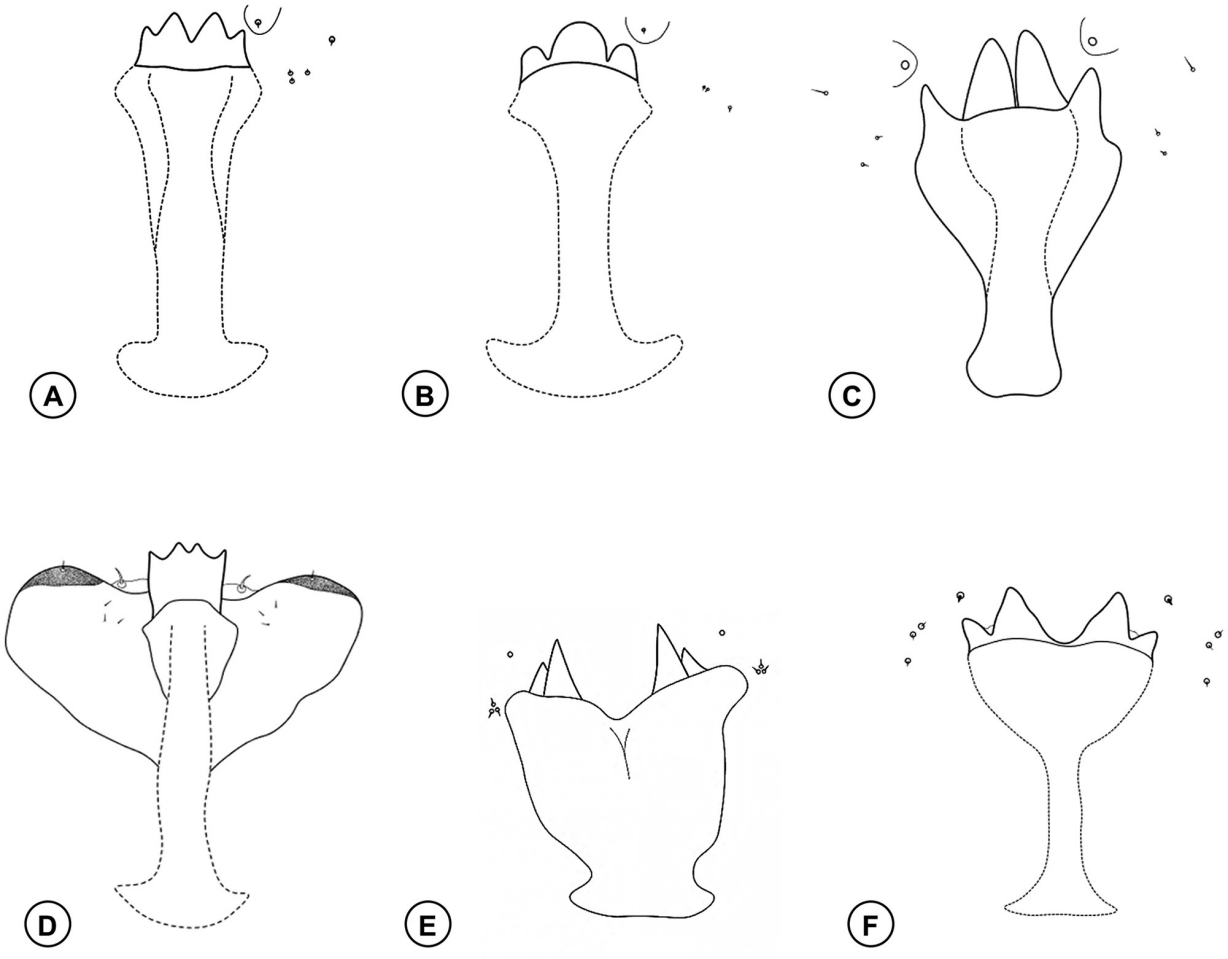
Larval prothoracic spatula. **(A)** *Bruggmanniella braziliensis* Tavares. **(B)** *Bruggmanniella oblita* Tavares. **(C)** *Bruggmanniella maytenuse* (Maia and Couri). **(D)** *Bruggmanniella perseae* Gagné. **(E)** *Bruggmanniella doliocarpi* Maia. **(F)** *Bruggmanniella miconia* **sp. nov.** Illustrations modified from Möhn [39], Gagné et al. [41] and Maia et al. [42].

1. Prothoracic spatula with 4 apical teeth (Fig 7A) ……………………………………………………………………………………………… 2

1a. Prothoracic spatula with 3 apical teeth (Fig 7B) ……………………………………………………………………………………………… 3

2. Teeth of prothoracic spatula of the same size (Fig 7D) ……………………………………………………………………… *B*. *perseae* Gagné

2a. Teeth of prothoracic spatula of different size (Fig 7E) …………………………………………………………………………………………4

3. Papillae of the terminal absent, stem base of prothoracic spatula of larva with lateral projection well developed (Fig 7B) …………………… *B*. *oblita* Tavares

3a. Papillae of the terminal present, stem base of prothoracic spatula of larva with lateral projection undeveloped (Fig 7C) …………………… *B*. *bumeliae* (Felt)

4. Inner teeth of prothoracic spatula separated from each other (Fig 7E) ………………………………………………………………………… 5

4a. Prothoracic spatula with inner teeth close to each other (Fig 7C) …………………………………………………………………………… 6

5. Inner and outer teeth with pointed apex, stem wide and short (Fig 7C) ……………………………………………………………………… *B*. *doliocarpi* Maia

5a. Inner and outer teeth with rounded apex, stem narrow and long (Fig 7D) …………………………………………………… *B*. *miconia* **sp. nov.**

6. Papillae of the terminal segment absent ……………………………………………………………………………………… *B*. *ingae* Urso-Guimarães and Amorim

6a. Papillae of the terminal segment present ……………………………………………………………………………………………… 7

7. Stem base of prothoracic spatula of larva without lateral projection ……………………………………………… *B*. *maytenuse* (Maia and Couri)

7a. Stem base of prothoracic spatula of larva with lateral projection …………………………………………………………………………… *B*. *braziliensis* Tavares

### Identification key to the known pupae of *Bruggmanniella*

**Fig 8:**
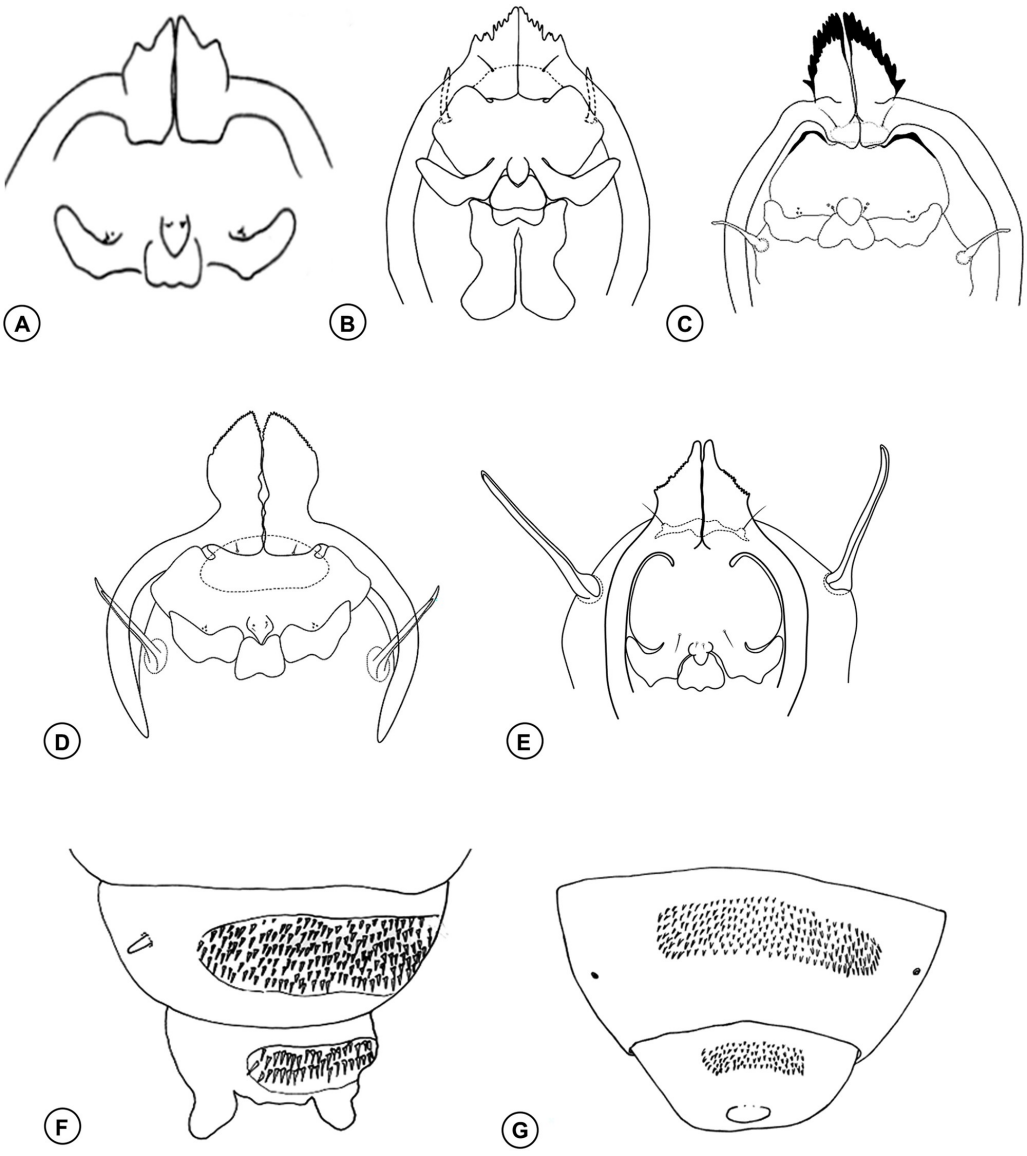
Pupal head and abdominal segments. **(A)** *Bruggmanniella bumeliae* (Felt). **(B)** *Bruggmanniella ingae* Urso-Guimarães and Amorim. **(C)** *Bruggmanniella byrsonimae* (Maia and Couri). **(D)** *Bruggmanniella duguetiae* Urso-Guimarães and Amorim. **(E)** *Bruggmanniella perseae* Gagné. **(F)** *Bruggmanniella byrsonimae* (Maia and Couri). **(G)** *Bruggmanniella ingae* Urso-Guimarães and Amorim. Illustrations modified from Urso-Guimarães and Amorim [24], Gagné [11], Gagné et al. [41], and Maia et al. [42].

1. Antennal horn with smooth outer edge (Fig 8A), projection at the base of the antennal horn present, abdominal dorsal spines inconspicuous …………………………… *B*. *bumeliae* (Felt)

1a. Antennal horn with serrated outer edge (Fig 8B), projection at the base of the antennal horn absent, abdominal dorsal spines conspicuous ……………………………………… 2

2. Antennal horn with smooth inner edge (Fig 8C) ……………………………………………………………………………… 3

2a. Antennal horn with serrated inner edge (Fig 8D) …………………………………………………………………………… 4

3. Antennal horn triangular, twice as long as the widest diameter, straight laterally except for serrations on the outer edge; upper facial margin thickened (Fig 8C); prothoracic spiracle shortened (0.3 mm length); dorsal spines conspicuous and gathered; projections absent in the eighth abdominal segment ………………………………………*B*. *doliocarpi* Maia

3a. Antennal horn twice as long as the widest diameter, convex laterally (Fig 8D); non-thickened upper facial margin; prothoracic spiracle elongated (0.6 mm length); dorsal spines conspicuous and shattered; projections present in the eighth abdominal segment (Fig 8F) ……………………………………… *B*. *duguetiae* Urso-Guimarães and Amorim

4. Upper facial margin thickened (Fig 8C) ……………………………………………………………………………… 5

4a. Upper facial margin not thickened …………………………………………………………………………………… 6

5. Antennal horn deeply serrated (Fig 8B); projections present in the seventh and eighth abdominal segments (Fig 8F) ……………………… *B*. *byrsonimae* (Maia and Couri)

5a. Antennal horn slightly serrated (Fig 8D); projections absent in the seventh and eighth abdominal segments …………………………………………*B*. *maytenuse* (Maia and Couri)

6. Abdominal spiracles rounded (Fig 8G); two pairs of lower facial papillae, two pairs of lower lateral papillae ……………………….…… *B*. *ingae* Urso-Guimarães and Amorim

6a. Abdominal spiracles spiniform (Fig 8F); other features not as above …………………………………… 7

7. Dorsal spines on the eighth abdominal segment present …………………………………………………… 8

7a. Dorsal spines on the eighth abdominal segment absent …………………………………………………… 9

8. Antennal horn short, approximately, as long as the widest diameter; one pair of lower facial setose papillae, two pairs of lower lateral papillae (one setose and one bare) ………………………… *B*. *oblita* Tavares

8a. Antennal horn long, approximately twice as long as the widest diameter; two pairs of lower facial papillae (one setose and one bare), three pairs of lower lateral papillae (one setose and two bare) ………… *B*. *braziliensis* Tavares

9. Antennal horn convex laterally (Fig 8E); one pair of lower facial setose papillae, lower lateral papillae absent; prothoracic spiracle very long (0.5 mm length) ………………………………………….……….…………… *B*. *perseae* Gagné

9a. Antennal horn straight laterally except for serrations on the outer edge; two pairs of lower facial papillae (one setose and one bare), two pairs of lower lateral setose papillae; prothoracic spiracle short (0.2 mm length) ……………………*B*. *miconia* **sp. nov.**

### Identification key to known adults of *Bruggmanniella*

1. Palpi with 1 segment ………………………………………… 2

1a. Palpi with 2 or more segments ……………………………… 3

2. Hypoproct deeply bilobed; male or female flagellomeres short and rounded, with two appressed circumfila connected to one another; needle-like part of the ovipositor 1.6 times the length of the seventh sternite; globoid, reddish when young and brown when mature, and glabrous galls occurring in clusters on branches of *Miconia* cf. *cinnamomifolia* (Melastomataceae), Brazil ……………………………… *B*. *miconia* **sp. nov.**

2a. Hypoproct unilobed or slightly bilobed; other features not as above ………………………………………………3

3. Ovipositor piriform ………………………………………………………………4

3a. Ovipositor tubular ………………………………………………………………5

**Fig 9:**
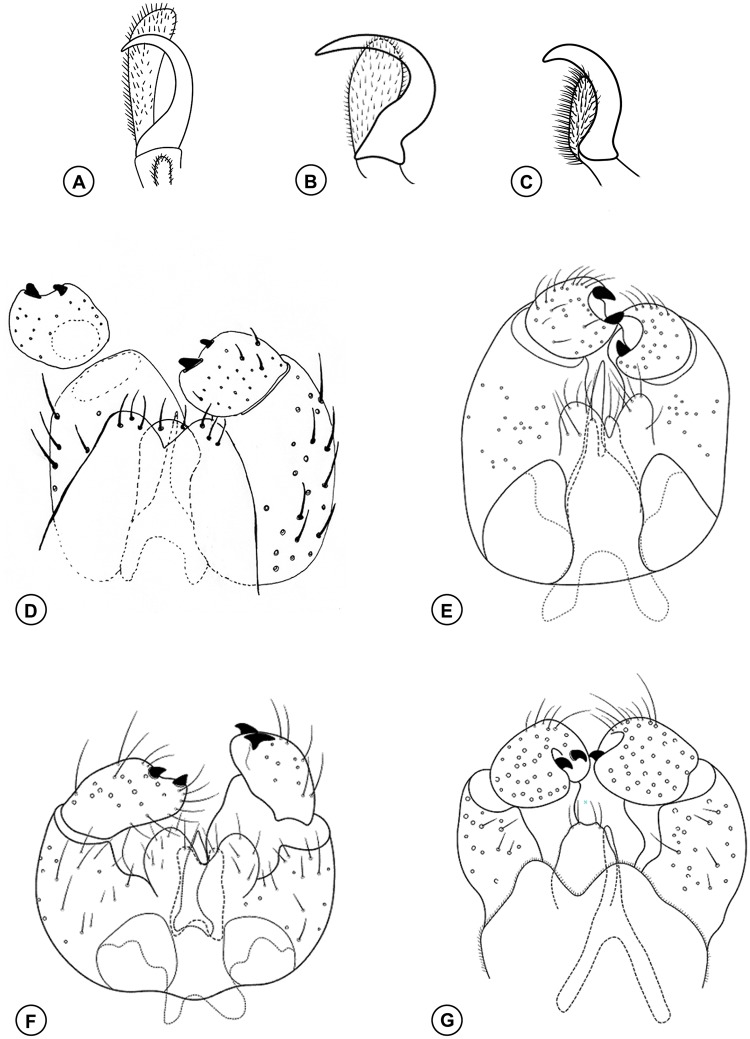
Terminalia and male tarsal claws. **(A)** *Bruggmanniella braziliensis* Tavares. **(B)** *Bruggmanniella doliocarpi* Maia. **(C)** *Bruggmanniella miconia* **sp. nov. (D)** *Bruggmanniella ingae* Urso-Guimarães and Amorim. **(E)** *Bruggmanniella doliocarpi* Maia. **(F)** *Bruggmanniella bumeliae* (Felt). **(G)** *Bruggmanniella byrsonimae* (Maia and Couri). Illustrations modified from Urso-Guimarães and Amorim [24], Möhn [39] and Maia et al. [42].

4. Empodium longer than claw (Fig 9A); needle-like part 1.8 times the length of the seventh sternite; hypoproct unilobed; male cerci slightly bilobed and longer than hypoproct; globoid, hairy and multichambered galls on swollen stem apices of *Sorocea bonplandii* (Moraceae), Brazil ………………………………*B*. *braziliensis* Tavares

4a. Empodium as long as claw (Fig 9B); needle-like part four times the length of the seventh sternite; male unknown; fusiform, brown and glabrous galls on stems of *Duguetiae furfuracea* (Annonaceae), Brazil …………………………………………… *B*. *duguetiae* Urso-Guimarães and Amorim

5. Cerci longer than hypoproct (Fig 9B) ……………………………… 6

5a. Cerci shorter or as long as hypoproct (Fig 9D) ……………………………… 7

6. Gonocoxite cylindrical, three times the length of gonostylus (Fig 9E); aedeagus longer than cerci; needle-like part of the ovipositor 1.5–1.7 times the length of the seventh sternite; fusiform, reddish and hairy galls on stems of *Doliocarpus dentatus* (Dilleniaceae), Brazil ……………………………… *B*. *doliocarpi* Maia

6a. Gonocoxite spread, twice as long as the length of gonostylus (Fig 9F); aedeagus as long as cerci; needle-like part of the ovipositor three times the length of the seventh sternite; fusiform galls on stems of *Bumelia lanuginosa* (Sapotaceae), USA (southern), Mexico ……………………………… *B*. *bumeliae* (Felt)

7. Pulvilli present (Fig 9A) ……………………………… 8

7a. Pulvilli absent (Fig 9B) ……………………………… 9

8. Empodium as long as claw (Fig 9B); cerci deeply bilobed and approximately as long as hypoproct; galls on fruits of *Persea americana* (Lauraceae), Colombia and Costa Rica ……………………………………………………………………….… *B*. *perseae* Gagné

8a. Empodium shorter than claw (Fig 9C); male unknown; fusiform, glabrous and multichambered galls on stems of *Schinus* sp. (Anacardiaceae), Brazil ………………… *B*. *oblita* Tavares

9. Hypoproct bilobed (Fig 9E–9G) ……………………………… 10

9a. Hypoproct unilobed (Fig 9D) ……………………………… 11

10. Circumfila of male or female flagellomeres sinuous; needle-like part of ovipositor four times the length of the seventh sternite; clavate, green when young and brown when mature, single or multichambered galls on swollen buds of *Byrsonima sericea* (Malpighiaceae), Brazil ……………………………… *B*. *byrsonimae* (Maia and Couri)

10a. Circumfila of male or female flagellomeres not sinuous; other features not as above ……………………………… 11

11. Hypoproct with straight apex; needle-like part of ovipositor 2.7 times the length of the seventh sternite; globoid, reddish and glabrous galls on modified fruits of *Maytenus obtusifolia* (Celastraceae), Brazil ……………………………… *B*. *maytenuse* (Maia and Couri)

11a. Hypoproct with rounded apex; needle-like part of the ovipositor six times the length of the seventh sternite; multichambered, green and hairy galls on ovary of *Inga edulis* (Fabaceae), Brazil ……………………………… *B*. *ingae* Urso-Guimarães and Amorim

## Conclusion

This is the first phylogenetic study of the genus *Bruggmanniella*. The results corroborated previous ideas about the relationship among *Bruggmanniella*, *Pseudasphondylia*, and *Illicomyia*. Some new nomenclatural combinations were proposed and supported by the results of our cladistic analyses: *Pseudasphondylia actinodaphnes*, *Pseudasphondylia cinnamomi*, and *Odontokeros* **gen. nov.** *Bruggmanniella* is a monophyletic genus with ten species distributed in the New World, including *Bruggmanniella byrsonimae* which was not added to the analysis. Further studies and sampling efforts should be conducted in order to find the missing stages of *B*. *byrsonimae* and test its placement among the genus. As we have indicated, studies on coevolution also need to be conducted to investigate the association of these genera with their host plants, as well as a biogeographic approach of the tribe Asphondyliini is still lacking.

## Supporting information

S1 Table Examined material. (DOCX)

S2 Table Normalized data. (DOCX)

S3 Table Matrix. (DOCX)

S1 File Input matrix script. (DOCX)

S1 Fig Tree under equal weight. (DOCX)

S2 Fig Tree under implied weight. (DOCX)

## References

[1] Gagné RJ, Jaschhof M. A Catalog of the Cecidomyiidae (Diptera) of the World. 4th ed Washington, DC, USA Entomological Society of Washington 2017.

[2] Borkent A, Brown B, Adler PH, Amorim DDS, Barber K, Bickel D, et al Remarkable fly (Diptera) diversity in a patch of Costa Rican cloud forest. Zootaxa. 2018;4402:053–090. 10.11646/zootaxa.4402.1.3 29690278

[3] Brown BV, Borkent A, Adler PH, Amorim DDS, Barber K, Bickel D, et al Comprehensive inventory of true flies (Diptera) at a tropical site. Communications biology. 2018;1:21 10.1038/s42003-018-0022-x 30271908 PMC6123690

[4] Hebert PD, Ratnasingham S, Zakharov EV, Telfer AC, Levesque-Beaudin V, Milton MA, et al Counting animal species with DNA barcodes: Canadian insects. Philosophical Transactions of the Royal Society B: Biological Sciences. 2016;371:150333. 10.1098/rstb.2015.0333 PMC4971185 27481785

[5] Amorim DDS, Rindal E. Phylogeny of the Mycetophiliformia, with proposal of the subfamilies Heterotrichinae, Ohakuneinae, and Chiletrichinae for the Rangomaramidae (Diptera, Bibionomorpha). Zootaxa. 2007;1535:1–92.

[6] Ševčík J, Kaspřák D, Mantič M, Fitzgerald S, Ševčíková T, Tóthová A, et al (2016). Molecular phylogeny of the megadiverse insect infraorder Bibionomorpha sensu lato (Diptera). PeerJ. 2016;4:1–30. 10.7717/peerj.2563 PMC5075709 27781163

[7] Wood DM, Borkent A. Phylogeny and Classification of the Nematocera In: McAlpine JF, Peterson BV, Shewell GE, Teskey HJ, Vockeroth JR, Wood DM, eds. Manual of Nearctic Diptera. Ottawa: Research Branch, Agriculture Canada 1989;27:1333–1370.

[8] Oosterbroek P, Courtney G. Phylogeny of the nematocerous families of Diptera (Insecta) Zoological Journal of the Linnean Society. 1995;115:267–311.

[9] Sikora T, Jaschhof M, Kasprak D, Mantic M., Sevcik J. Considerable congruence, enlightening conflict: molecular analysis largely supports morphology-based hypotheses on Cecidomyiidae (Diptera) phylogeny. Zoological Journal of the Linnean Society. 2019;185:98–110.

[10] Dorchin N, Harris KM, Stireman JO. Phylogeny of the gall midges (Diptera, Cecidomyiidae, Cecidomyiinae): Systematics, evolution of feeding modes and diversification rates. Molecular Phylogenetics and Evolution. 2019;140:106602 10.1016/j.ympev.2019.106602 31449853

[11] Gagné RJ. The gall midges of the Neotropical Region. Cornell University Press 1994

[12] Lin Sheng-Feng, Tokuda M, Yang Man-Miao. The first record of genus *Bruggmanniella* (Diptera: Cecidomyiidae) from Taiwan with description of a new species inducing bud galls on *Neolitsea parvigemma* (Lauraceae). Journal of Asia-Pacific Entomology. 2019;22:203–207.

[13] Mani MS. Ecology of Plant Galls. Junk, The Hague. 1964;434.

[14] Price PW, Fernandes GW, Waring GL. Adaptive nature of insect galls. Environ Entomol. 1987;16:15–24.

[15] Yukawa J, Rohfritsch O. Biology and ecology of gall-inducing Cecidomyiidae In: Raman A, Schaefer CW, Withers TM, editors. Biology and ecology of gall-inducing Cecidomyiidae. Science Publishers, Inc, New Hampshire 2005 p. 274–304.

[16] Tokuda M, Yang MM, Yukawa J. Taxonomy and molecular phylogeny of *Daphnephila* gall midges (Diptera: Cecidomyiidae) inducing complex leaf galls on Lauraceae, with descriptions of five new species associated with *Machilus thunbergii* in Taiwan. Zoological Science. 2008;25:533–546. 10.2108/zsj.25.533 18558807

[17] Urso-Guimarães MV, Scareli-Santos C. Galls and gall makers in plants from the Pé-de-Gigante Cerrado reserve, Santa Rita do Passa Quatro, SP, Brazil. Brazilian Journal of Biology. 2006;66(1B):357–369. 10.1590/s1519-69842006000200018 16710528

[18] Kovalev OV. A review of the gall-midges (Diptera, Itonididae) of the extreme south of the Soviet Far East. The supertribe Asphondyliidi. Entomological Review. 1964;43:215–228.

[19] Yukawa JA. Revision of the Japanese gall midges Memorial of Faculty of Agriculture, Kagoshima University.1971;8:1–203.

[20] Tokuda M, Yukawa J. Morphological features of the mature larva and pupa of *Pseudasphondylia rokuharensis* Monzen (Diptera: Cecidomyiidae). Esakia, 2002;42:11–17.

[21] Tokuda M, Yukawa J. Two new and three known Japanese species of genus *Pseudasphondylia* Monzen (Diptera: Cecidomyiidae: Asphondyliini) and their life history strategies. Annals of the Entomological Society of America. 2005;98:259–272.

[22] Tokuda M, Yukawa J. First records of genus *Bruggmanniella* (Diptera: Cecidomyiidae: Asphondyliini) from Palearctic and Oriental Regions, with descriptions of two new species that induce stem galls on Lauraceae in Japan. Annals of the Entomological Society of America. 2006;99:629–637.

[23] Tokuda M, Yukawa J. Biogeography and evolution of gall midges (Diptera: Cecidomyiidae) inhabiting broad-leaved evergreen forests in oriental and eastern palearctic regions. Oriental Insects. 2007;41:121–139.

[24] Urso-Guimarães MV, Amorim DDS. Two new species of *Bruggmanniella* Tavares, 1909 (Diptera, Cecidomyiidae) from Brazil. Zootaxa. 2005;11:429–436.

[25] Tokuda M. *Illiciomyia* Tokuda, a new genus for *Illiciomyia yukawai* sp. n. (Diptera: Cecidomyiidae: Asphondyliini) inducing leaf galls on *Illicium anisatum* (Illiciaceae) in Japan. Esakia. 2004;44:1–11.

[26] Goldenberg R, Almeda F, Caddah MK, Martins AB, Meirelles J, Michelangeli FA, et al, Nomenclator botanicus for the neotropical genus *Miconia* (Melastomataceae: Miconieae). Phytotaxa. 2013;106(1):1–171.

[27] Bacci LF, Caddah MK, Goldenberg R. The genus *Miconia* (Melastomataceae) in Espírito Santo, Brazil. Phytotaxa. 2016;271(1):1–92.

[28] Maia VC. New genera and species of gall midges (Diptera, Cecidomyiidae) from three restingas of Rio de Janeiro State, Brazil. Rev. Bras. Zool. 2001;18(1):1–32.

[29] Cumming JM, Wood DM. Adult morphology and terminology. Manual of Central American Diptera. 2009;1:9–50.

[30] Folmer O, Black M, Hoeh W, Lutz R, Vrijenhoek. DNA primers for amplification of mitochondrial cytochrome c oxidase subunit I from diverse metazoan invertebrates. Mol Mar Biol Biotech. 1994;3:294–299. 7881515

[31] Kearse M, Moir R, Wilson A, Stones-Havas S, Cheung M, Sturrock S, et al Geneious Basic: an integrated and extendable desktop software platform for the organization and analysis of sequence data. Bioinformatics. 2012;28:1647–1649. Available from: https://www.geneious.com/. 10.1093/bioinformatics/bts199 22543367 PMC3371832

[32] Thiele K. The holy grail of the perfect character: the cladistic treatment of morphometric data. Cladistics. 1993;9:275–304. 10.1111/j.1096-0031.1993.tb00226.x 34929957

[33] Goloboff PA, Carpenter JM, Arias JS, Esquivel DRM. Weighting against homoplasy improves phylogenetic analysis of morphological data sets. Cladistics. 2008;24:758–773.

[34] Goloboff PA, Farris JS, Nixon K. TNT: Tree analysis using New Technology. Version 1.1 [software]. http://www.lillo.org.ar/phylogeny/tnt/.

[35] Bremer K. Branch support and tree stability. Cladistics. 1994;10:295–304.

[36] Kluge AG, Farris JS. Quantitative phyletic and the evolution of anurans. Systematic Biology. 1969;18:1–32.

[37] Farris JS. The retention index and the rescaled consistency index. Cladistics. 1989;5:417–419. 10.1111/j.1096-0031.1989.tb00573.x 34933481

[38] Möhn E. Gallmucken (Diptera, Itonididae) aus El Salvador. 4. Zur Phylogenie der Asphondyliidi der neotropischen und holarktischen Region. Senckenbergiana Biologica. 1961;42:131–330. German.

[39] Möhn E. Studien über neotropische Gallmücken (Diptera, Itonididae).1.Teil. (Fortsetzung). Broteria, Série de Ciências Naturais. 1963;32:3–23. German.

[40] Tavares JS. Contributio prima ed cognitionem cecidologiae Braziliae. Brotéria, Série Zoológica. 1909;8:5–28. Latin.

[41] Gagné RJ, Posada F, Gil ZN. A new species of *Bruggmanniella* (Diptera: Cecidomyiidae) aborting young fruit of avocado, *Persea americana* (Lauraceae), in Colombia and Costa Rica. Proceedings of the Entomological Society of Washington. 2004:1063:547–553.

[42] Maia VC, Couri MS, Monteiro RF. Sobre seis espécies de Asphondylia Loew, 1850 do Brasil (Diptera, Cecidomyiidae). Revista Brasileira de Entomologia. 1992;36:653–661.

