# Supplementary material for: Cladistic analysis of the genus Bruggmanniella Tavares (Diptera, Cecicomyiidae, Asphondyliini) with evolutionary inferences on the gall inducer-host plant association and description of a new Brazilian species
Source: PLoS One. 2020 Feb 5;15(2):e0227853. doi: 10.1371/journal.pone.0227853 (PMC7001989; doi:10.1371/journal.pone.0227853)
Supplement: S1 Table — (DOCX) [file pone.0227853.s001.docx]

**S1 Table – Examined Material**

All examined specimens are deposited in KUEC, Kyushu University, Fukuoka; SMNS, Stuttgart, Germany; SMNH, Washington DC, USA; MZSP, São Paulo, Brazil; MNRJ, Rio de Janeiro, Brazil; INRA, Versailles, France.

| Outer-group | Types examined | Total of examined specimens | Distribution | Type depositories |
| --- | --- | --- | --- | --- |
| *Lopesia andirae* Garcia, Lima, Calado and Urso-Guimarães, 2017 | Holotype 1♂, Paratype (1♂, 2♀, 10 larvae, 3 pupae) | 17 | Neotropical | MZSP |
| *Asphondylia canastrae* Urso-Guimarães and Amorim, 2002 | Holotype 1♂, Paratype (1♀, 1 larva, 5 pupae) | 8 | Neotropical | MZSP |
| *Asphondylia peplonia* Maia, 2001 | Literature | ______ | Neotropical | MNRJ |
| *Asphondylia sanctipetri* Urso-Guimarães and Amorim, 2002 | Holotype 1♂, Paratype (1♀, 1 larva, 2 pupae) | 5 | Neotropical | MZSP |
| *Bruggmannia acaudata* Maia, 2004 | Literature | ______ | Neotropical | MNRJ |
| *Illiciomyia yukawai* Tokuda, 2004 | Literature | ______ | Oriental | KUEC |
| *Parazalepidota clusiae* Maia, 2001 | Holotype 1♂, Paratype (1♀, 1 larva, 1 pupa) | 4 | Neotropical | MNRJ |
| *Pseudasphondyia elaeocarpi* Tokuda and Yukawa, 2005 | Literature/Photos | ______ | Oriental, Palearctic | KUEC |
| *Pseudasphondylia kiritanii* Tokuda and Yukawa, 2005 | Literature/Photos | ______ | Palearctic | KUEC |
| *Pseudasphondylia matatabi* Yuasa and Kumazawa, 1938 | Literature/Photos | ______ | Palearctic | KUEC |
| *Pseudasphondylia neolitseae* Yukawa, 1974 | Literature/Photos | ______ | Palearctic | KUEC |
| *Pseudasphondylia rauwolfiae* Coutin, 1980 | Literature/Photos | ______ | Australia | INRA |
| *Pseudasphondylia rokuharensis* Monzen, 1955 | Literature/Photos | ______ | Oriental, Palearctic | SMNH |
| *Schizomyia macrocapillata* Maia, 2005 | Literature/Photos | ______ | Neotropical | MNRJ |

| Inner-group | Types examined | Total of examined specimens | Distribution | Type depositories |
| --- | --- | --- | --- | --- |
| *Bruggmanniella actinodaphnes* Tokuda and Yukawa, 2006 | Literature/Photos | ______ | Palearctic | KUEC |
| *Bruggmanniella braziliensis* Tavares, 1909 | Literature | ______ | Neotropical | SMNS |
| *Bruggmanniella brevipes* Lin, Yang and Tokuda, 2018 | Literature/Photos | ______ | Oriental | KUEC |
| *Bruggmanniella bumeliae* (Felt, 1907) Gagné, 1994 | Holotype 1♂, Paratype (1♀) | 2 | Nearctic | SMNH |
| *Bruggmanniella byrsonimae* (Maia and Couri, 1992) | Holotype 1♂, Paratype (1♂, 5♀, 5 pupae) | 12 | Neotropical | MNRJ |
| *Bruggmanniella cinnamomi* Tokuda and Yukawa, 2006 | Literature/Photos | ______ | Oriental | KUEC |
| *Bruggmanniella doliocarpi* Maia, 2010 | Holotype 1♂, Paratype (2♂, 2♀, 2 larvae) | 7 | Neotropical | MNRJ |
| *Bruggmanniella duguetiae* Urso-Guimarães and Amorim, 2005 | Holotype 1♀, Paratype (3 pupae) | 4 | Neotropical | MZSP |
| *Bruggmanniella ingae* Urso-Guimarães and Amorim, 2005 | Holotype 1♂, Paratype (1♂, 3♀, 17 pupa, 2 larvae) | 24 | Neotropical | MZSP |
| *Bruggmanniella maytenuse* (Maia and Couri, 1992) | Holotype 1♀, Paratype (1♂, 3♀, 3 larvae, 1 exuvia) | 9 | Neotropical | MNRJ |
| *Bruggmanniella oblita* Tavares, 1920 | Literature | ______ | Neotropical | SMNS |
| *Bruggmanniella perseae* Gagné, 2004 | Paratype (2♂, 2♀, 7 larvae, 5 pupae) | 16 | Neotropical | SMNH |
| *Bruggmanniella miconia* Garcia, Lamas and Urso-Guimarães sp. n | Holotype 1♀, Paratype (6 larvae, 10 pupae) | 17 | Neotropical | MZSP |
