# Supplementary material for: Cladistic analysis of the genus Bruggmanniella Tavares (Diptera, Cecicomyiidae, Asphondyliini) with evolutionary inferences on the gall inducer-host plant association and description of a new Brazilian species
Source: PLoS One. 2020 Feb 5;15(2):e0227853. doi: 10.1371/journal.pone.0227853 (PMC7001989; doi:10.1371/journal.pone.0227853)
Supplement: S2 Table — (DOCX) [file pone.0227853.s002.docx]

**S2 Table –** **Normalized Data**

The table below shows the two normalized continuous characters. The left column shows the raw data in mm and the right column shows the normalized data.

| *Raw data (mm)* | | | | *Normalized data* | | |
| --- | --- | --- | --- | --- | --- | --- |
| Taxa | **0** | **1** |  | **Taxa** | **0** | **1** |
| *Lopesia_andirae* | 0.12 | 0.06 |  | *Lopesia_andirae* | 0.09 | 0.46 |
| *Bruggmannia_acaudata* | 0.07 | 0.12 |  | *Bruggmannia_acaudata* | 0.00 | 0.92 |
| *Schizomyia_macrocapillata* | 0.14 | 0.1 |  | *Schizomyia_macrocapillata* | 0.13 | 0.77 |
| *Parazalepidota_clusiae* | 0.26 | 0.06 |  | *Parazalepidota_clusiae* | 0.36 | 0.46 |
| *Asphondylia_anthocercidis* | 0.24 | 0.06 |  | *Asphondylia_anthocercidis* | 0.32 | 0.46 |
| *Asphondylia_canastrae* | 0.6 | 0.04 |  | *Asphondylia_canastrae* | 1.00 | 0.31 |
| *Asphondylia_peploniae* | 0.2 | 0.03 |  | *Asphondylia_peploniae* | 0.25 | 0.23 |
| *Asphondylia_sanctipetri* | 0.16 | 0.04 |  | *Asphondylia_sanctipetri* | 0.17 | 0.31 |
| *Illiciomyia_yukawai* | 0.22 | 0.13 |  | *Illiciomyia_yukawai* | 0.28 | 1.00 |
| *Bruggmanniella_brevipes* | 0.37 | 0.04 |  | *Bruggmanniella_brevipes* | 0.57 | 0.31 |
| *Pseudasphondylia_elaeocarpi* | 0.22 | 0.07 |  | *Pseudasphondylia_elaeocarpi* | 0.28 | 0.54 |
| *Pseudasphondylia_neolitseae* | 0.35 | 0.07 |  | *Pseudasphondylia_neolitseae* | 0.53 | 0.54 |
| *Bruggmanniella_cinnamomi* | 0.47 | 0.08 |  | *Bruggmanniella_cinnamomi* | 0.75 | 0.62 |
| *Bruggmanniella_actinodaphnes* | 0.37 | 0 |  | *Bruggmanniella_actinodaphnes* | 0.57 | 0.00 |
| *Pseudasphondylia_matatabi* | 0.35 | 0.11 |  | *Pseudasphondylia_matatabi* | 0.53 | 0.85 |
| *Pseudasphondylia_kiritanii* | 0.2 | 0.08 |  | *Pseudasphondylia_kiritanii* | 0.25 | 0.62 |
| *Pseudasphondylia_rokuharensis* | 0.25 | 0.08 |  | *Pseudasphondylia_rokuharensis* | 0.34 | 0.62 |
| *Pseudasphondylia_rauwolfiae* | ? | ? |  | *Pseudasphondylia_rauwolfiae* | ? | ? |
| *Bruggmanniella_*sp.n | 0.35 | 0.06 |  | *Bruggmanniella_***sp.n** | 0.53 | 0.46 |
| *Bruggmanniella_perseae* | 0.37 | 0.09 |  | *Bruggmanniella_perseae* | 0.57 | 0.69 |
| *Bruggmanniella_duguetiae* | 0.6 | 0.07 |  | *Bruggmanniella_duguetiae* | 1.00 | 0.54 |
| *Bruggmanniella_doliocarpi* | 0.44 | 0.05 |  | *Bruggmanniella_doliocarpi* | 0.70 | 0.38 |
| *Bruggmanniella_bumeliae* | ? | ? |  | *Bruggmanniella_bumeliae* | ? | ? |
| *Bruggmanniella_maytenuse* | 0.35 | ? |  | *Bruggmanniella_maytenuse* | 0.53 | ? |
| *Bruggmanniella_ingae* | 0.3 | 0.02 |  | *Bruggmanniella_ingae* | 0.43 | 0.15 |
| *Bruggmanniella_oblita* | 0.13 | 0.08 |  | *Bruggmanniella_oblita* | 0.11 | 0.62 |
| *Bruggmanniella_braziliensis* | 0.29 | 0.09 |  | *Bruggmanniella_braziliensis* | 0.42 | 0.69 |
