# Supplementary material for: Cladistic analysis of the genus Bruggmanniella Tavares (Diptera, Cecicomyiidae, Asphondyliini) with evolutionary inferences on the gall inducer-host plant association and description of a new Brazilian species
Source: PLoS One. 2020 Feb 5;15(2):e0227853. doi: 10.1371/journal.pone.0227853 (PMC7001989; doi:10.1371/journal.pone.0227853)
Supplement: S3 Table — (DOCX) [file pone.0227853.s003.docx]

**S3 Table – Matrix**

| Taxa | 2 | 3 | 4 | 5 | 6 | 7 | 8 | 9 | 10 | 11 | 12 | 13 | 14 | 15 | 16 | 17 | 18 | 19 | 20 | 21 | 22 | 23 | 24 | 25 | 26 | 27 | 28 | 29 | 30 |
| --- | --- | --- | --- | --- | --- | --- | --- | --- | --- | --- | --- | --- | --- | --- | --- | --- | --- | --- | --- | --- | --- | --- | --- | --- | --- | --- | --- | --- | --- |
| *Lopesia_andirae* | 0 | 0 | 0 | 0 | 0 | 0 | 0 | 0 | 0 | 0 | 0 | 0 | 0 | 0 | 0 | 0 | 0 | 0 | 0 | - | 0 | 0 | 0 | 0 | 0 | 0 | 0 | 0 | 0 |
| *Bruggmannia_acaudata* | 1 | - | - | - | - | - | 0 | 0 | 0 | 0 | 0 | 0 | 0 | 0 | 0 | 1 | 0 | 0 | 1 | 1 | 1 | 0 | 1 | 0 | 1 | 1 | 1 | 0 | 1 |
| *Schizomyia_macrocapillata* | 0 | 0 | 0 | 0 | 0 | 0 | 0 | 1 | 0 | 0 | 0 | 0 | 0 | 0 | 0 | 1 | 0 | 1 | 1 | 0 | 0 | 0 | 1 | 0 | 1 | 1 | 0 | 0 | 0 |
| *Parazalepidota_clusiae* | 0 | 2 | 2 | 0 | 2 | 2 | ? | 1 | 0 | 0 | 0 | 0 | 0 | 1 | 1 | 0 | 0 | 1 | 1 | 1 | 0 | 0 | 1 | 0 | 1 | 1 | 0 | 0 | 0 |
| *Asphondylia_anthocercidis* | 0 | 2 | 1 | 1 | 1 | 0 | 0 | 0 | 0 | 0 | 0 | 1 | 1 | 0 | 0 | 1 | 0 | 1 | 1 | 0 | 1 | 0 | 1 | 0 | 1 | 1 | 0 | 0 | 0 |
| *Asphondylia_peploniae* | 0 | 0 | 0 | 1 | 1 | 2 | 0 | 0 | 0 | 0 | 0 | 0 | 1 | 0 | 0 | 1 | 0 | 1 | 1 | 0 | 1 | 1 | 1 | 0 | 1 | 1 | 0 | 0 | 0 |
| *Asphondylia_sanctipetri* | 0 | 2 | 1 | 1 | 1 | 2 | 0 | 0 | 0 | 0 | 1 | 0 | 1 | 0 | 0 | 1 | 0 | 1 | 1 | 0 | 1 | 0 | 1 | 0 | 1 | 1 | 0 | 0 | 0 |
| *Asphondylia_canastrae* | 0 | 2 | 0 | 1 | 1 | 2 | ? | 0 | 0 | 0 | 1 | 1 | 1 | 0 | 0 | 1 | 0 | 1 | 1 | 0 | 1 | 0 | 1 | 0 | 1 | 1 | 0 | 0 | 0 |
| *Illiciomyia_yukawai* | 0 | 1 | 2 | 0 | 0 | 2 | 1 | 1 | 0 | 1 | 0 | 0 | 0 | 0 | 0 | 1 | 1 | 1 | 1 | 1 | 0 | 0 | 1 | 1 | 1 | 1 | 0 | 1 | 1 |
| *Bruggmanniella_brevipes* | 0 | 2 | 1 | 1 | 0 | 1 | 1 | 0 | 0 | 2 | 0 | 0 | 0 | 0 | 0 | 1 | 1 | 1 | 1 | ? | 0 | 0 | 1 | 0 | 1 | 1 | 0 | 0 | 1 |
| *Pseudasphondylia_elaeocarpi* | 0 | 2 | 2 | 1 | 2 | 1 | 1 | 0 | 0 | 1 | 0 | 0 | 0 | 0 | 0 | 1 | 1 | 1 | 1 | 1 | 0 | 0 | 1 | 0 | 1 | 1 | 0 | 1 | 1 |
| *Pseudasphondylia_neolitseae* | 0 | 0 | 0 | 0 | 1 | 1 | 0 | 0 | 0 | 1 | 0 | 0 | 0 | 0 | 0 | 1 | 1 | 1 | 1 | 1 | 0 | 0 | 1 | 0 | 1 | 1 | 1 | 1 | 1 |
| *Bruggmanniella_cinnamomi* | 0 | 0 | 0 | 1 | 1 | 1 | 1 | 0 | 1 | 0 | 0 | 0 | 0 | 1 | 1 | 1 | 1 | 1 | 1 | 1 | 0 | 0 | 1 | 0 | 1 | 1 | 0 | 0 | 1 |
| *Bruggmanniella_actinodaphnes* | 0 | 0 | 0 | 1 | 1 | 1 | 1 | 0 | 0 | 2 | 0 | 0 | 0 | 0 | 0 | 1 | 1 | 1 | 1 | 1 | 0 | 0 | 1 | 0 | 1 | 1 | 0 | 0 | 1 |
| *Pseudasphondylia_matatabi* | 0 | 2 | 2 | 1 | 2 | 1 | 0 | 0 | 0 | 1 | 0 | 0 | 0 | 0 | 0 | 1 | 1 | 1 | 1 | 1 | 0 | 0 | 1 | 0 | 1 | 1 | 0 | 1 | 0 |
| *Pseudasphondylia_kiritanii* | 0 | 2 | 2 | 1 | 2 | 1 | 0 | 0 | 0 | 1 | 0 | 0 | 0 | 0 | 0 | 1 | 1 | 1 | 1 | 1 | 0 | 0 | 1 | 0 | 1 | 1 | 0 | 0 | 1 |
| *Pseudasphondylia_rokuharensis* | 0 | 2 | 2 | 0 | 2 | 1 | 0 | 0 | 0 | 1 | 0 | 0 | 0 | 0 | 0 | 1 | 1 | 1 | 1 | 1 | 0 | 0 | 1 | 0 | 1 | 1 | 0 | 1 | 1 |
| *Pseudasphondylia_rauwolfiae* | 0 | 2 | 2 | 0 | 2 | 1 | ? | ? | ? | ? | ? | ? | ? | ? | ? | ? | ? | ? | ? | ? | ? | ? | 1 | 0 | 1 | 1 | 0 | 1 | 0 |
| *Bruggmanniella_miconia_*sp.n | 0 | 2 | 2 | 1 | 2 | 1 | 1 | 0 | 0 | 1 | 0 | 1 | 0 | 0 | 0 | 1 | 1 | 1 | 1 | 1 | 0 | 0 | 1 | 0 | 1 | 1 | 0 | 0 | 0 |
| *Bruggmanniella_perseae* | 0 | 2 | 2 | 1 | 2 | 1 | 1 | 0 | 0 | 1 | 0 | 1 | 0 | 1 | 0 | 1 | 1 | 1 | 1 | 1 | 0 | 0 | 1 | 0 | 1 | 1 | 0 | 0 | 0 |
| *Bruggmanniella_duguetiae* | ? | ? | ? | ? | ? | ? | ? | 0 | 0 | 1 | 1 | 1 | 0 | 0 | 0 | 1 | 1 | 1 | 1 | 0 | 0 | 1 | 1 | 0 | ? | ? | ? | ? | 0 |
| *Bruggmanniella_doliocarpi* | 0 | 2 | 2 | 1 | 2 | 1 | 1 | 0 | 0 | 1 | 1 | 0 | 0 | 0 | 0 | 1 | 1 | 1 | 1 | 1 | 0 | 1 | 1 | 0 | 1 | 1 | 0 | 0 | 0 |
| *Bruggmanniella_bumeliae* | 0 | 1 | 2 | 1 | 2 | 1 | 0 | 0 | 0 | 2 | 0 | 1 | 0 | 0 | 0 | 1 | 1 | 1 | 1 | 0 | 0 | 0 | 1 | 0 | 1 | 1 | 0 | 0 | 0 |
| *Bruggmanniella_maytenuse* | 0 | 2 | 2 | 0 | 2 | 1 | 0 | 0 | 0 | 1 | 0 | 1 | 0 | 0 | 0 | 1 | 1 | 1 | 1 | 1 | 0 | 0 | 1 | 0 | ? | ? | ? | ? | 0 |
| *Bruggmanniella_ingae* | 0 | 2 | 2 | 1 | 2 | 1 | 0 | 0 | 0 | 2 | 0 | 1 | 0 | 0 | 0 | 1 | 0 | 1 | 1 | 1 | 0 | 0 | 1 | 0 | 1 | 1 | 0 | 0 | 0 |
| *Bruggmanniella_oblita* | 0 | 1 | 2 | 1 | 2 | 1 | 1 | 1 | 0 | 1 | 0 | 1 | 0 | 0 | 0 | 1 | 1 | 1 | 0 | 1 | ? | ? | 1 | 0 | 1 | 1 | ? | 0 | 0 |
| *Bruggmanniella_braziliensis* | 0 | 2 | 2 | 1 | 2 | 1 | 0 | 0 | 0 | 1 | 0 | 1 | 0 | 0 | 0 | 1 | 1 | 1 | 0 | ? | 0 | 0 | 1 | 0 | 1 | 1 | 0 | 0 | 0 |

**Continue….**

| Taxa |  | 31 | 32 | 33 | 34 | 35 | 36 | 37 | 38 | 39 | 40 | 41 | 42 | 43 | 44 | 45 | 46 | 47 | 48 | 49 | 50 | 51 | 52 | 53 | 54 | 55 | 56 |
| --- | --- | --- | --- | --- | --- | --- | --- | --- | --- | --- | --- | --- | --- | --- | --- | --- | --- | --- | --- | --- | --- | --- | --- | --- | --- | --- | --- |
| *Lopesia_andirae* |  | 0 | 0 | 0 | 0 | 0 | 0 | 0 | 0 | 0 | 0 | 0 | 0 | 0 | 0 | 0 | 0 | 0 | 0 | 0 | 0 | 0 | 0 | 0 | 0 | 0 | 0 |
| *Bruggmannia_acaudata* |  | 1 | 1 | 1 | ? | 1 | 0 | 0 | 1 | 1 | 0 | 1 | 2 | 1 | - | 1 | 1 | 3 | 0 | 0 | 0 | 1 | 1 | ? | 0 | 0 | 0 |
| *Schizomyia_macrocapillata* |  | 0 | 1 | 0 | 1 | 1 | 0 | 0 | 1 | 1 | 1 | 1 | 2 | 1 | - | 1 | 1 | 3 | 0 | 0 | 0 | 1 | 1 | 1 | 0 | 0 | 0 |
| *Parazalepidota_clusiae* |  | 1 | 1 | 2 | 0 | 1 | 2 | 0 | 1 | 1 | 1 | 0 | 1 | 0 | 0 | 1 | 0 | 2 | 0 | 0 | 0 | 1 | 1 | 1 | 1 | 1 | 0 |
| *Asphondylia_anthocercidis* |  | 0 | 1 | 1 | 0 | 1 | 1 | 0 | 0 | 1 | 1 | 0 | 1 | 0 | 0 | 0 | 0 | 1 | 0 | 0 | 0 | 1 | 1 | 1 | 1 | 1 | 3 |
| *Asphondylia_peploniae* |  | 0 | 1 | 1 | 0 | 1 | 1 | 0 | 0 | 1 | 1 | 1 | 1 | 0 | 0 | 0 | 1 | 3 | 0 | 0 | 0 | 1 | 1 | 1 | 1 | 1 | 2 |
| *Asphondylia_sanctipetri* |  | 0 | 1 | 1 | 0 | 1 | 0 | 0 | 0 | 1 | 1 | 0 | 1 | 0 | 0 | 0 | 0 | 1 | 0 | 0 | 1 | 1 | 1 | 1 | 1 | 1 | 1 |
| *Asphondylia_canastrae* |  | 0 | 1 | 1 | 0 | 1 | 0 | 0 | 0 | 1 | 1 | 0 | 1 | 0 | 0 | 0 | 0 | 1 | 0 | 0 | 1 | 1 | 1 | 1 | 1 | 1 | 2 |
| *Illiciomyia_yukawai* |  | 0 | 1 | 2 | 1 | 1 | 1 | 1 | 1 | 1 | 1 | 1 | 2 | 0 | 1 | 0 | 0 | 1 | 0 | 0 | 0 | 1 | 1 | 1 | 1 | 1 | 0 |
| *Bruggmanniella_brevipes* |  | 0 | 1 | 1 | 0 | 1 | 1 | 0 | 1 | 1 | 1 | 0 | 2 | 0 | 1 | 0 | 0 | 1 | 0 | 0 | 0 | 1 | 1 | 1 | 1 | 1 | 0 |
| *Pseudasphondylia_elaeocarpi* |  | 1 | 1 | 1 | 0 | 1 | 2 | 0 | 1 | 1 | 1 | 0 | 1 | 0 | 1 | 0 | 0 | 1 | 0 | 0 | 0 | 1 | 1 | 1 | 1 | 1 | 0 |
| *Pseudasphondylia_neolitseae* |  | 1 | 1 | 2 | 0 | 1 | 1 | 0 | 1 | 1 | 1 | 1 | 1 | 0 | 1 | 0 | 0 | 1 | 0 | 0 | 2 | 1 | 1 | 1 | 1 | 1 | 0 |
| *Bruggmanniella_cinnamomi* |  | 0 | 1 | 1 | 0 | 1 | 2 | 1 | 1 | 1 | 1 | 1 | 1 | 0 | 1 | 0 | 1 | 0 | 0 | 0 | 2 | 1 | 1 | 1 | 1 | 1 | 1 |
| *Bruggmanniella_actinodaphnes* |  | 0 | 1 | 2 | 0 | 1 | 1 | 1 | 1 | 1 | 1 | 1 | 1 | 0 | 1 | 0 | 1 | 1 | 0 | 0 | 1 | 1 | 1 | 1 | 1 | 1 | 1 |
| *Pseudasphondylia_matatabi* |  | 1 | 1 | 1 | 0 | 1 | 1 | 0 | 1 | 1 | 1 | 1 | 1 | 0 | 1 | 0 | 0 | 1 | 0 | 1 | 0 | 1 | 1 | 1 | 1 | 1 | 2 |
| *Pseudasphondylia_kiritanii* |  | 1 | 1 | 0 | 0 | 1 | 1 | 0 | 1 | 1 | 1 | 1 | 1 | 0 | 1 | 0 | 0 | 1 | 0 | 0 | 0 | 1 | 1 | 1 | 1 | 1 | 2 |
| *Pseudasphondylia_rokuharensis* |  | 1 | 1 | 0 | 0 | 1 | 1 | 0 | 1 | 1 | 1 | 1 | 1 | 0 | 1 | 0 | 0 | 0 | 0 | 2 | 0 | 1 | 1 | 1 | 1 | 1 | 3 |
| *Pseudasphondylia_rauwolfiae* |  | 0 | 1 | 0 | 0 | 1 | 0 | 0 | 1 | 1 | 1 | 1 | 1 | 0 | 1 | 0 | 0 | 1 | 0 | 0 | 1 | 1 | 1 | 1 | 1 | 1 | 3 |
| *Bruggmanniella_miconia_*sp.n |  | 0 | 1 | 3 | 0 | 1 | 0 | 0 | 0 | 1 | 1 | 0 | 1 | 0 | 1 | 0 | 0 | 1 | 0 | 0 | 0 | 1 | 1 | 1 | 1 | 1 | 1 |
| *Bruggmanniella_perseae* |  | 0 | 1 | 1 | 0 | 1 | 1 | 1 | 0 | 1 | 1 | 0 | 1 | 0 | 1 | 0 | 0 | 1 | 0 | 0 | 1 | 1 | 1 | 1 | 1 | 1 | 3 |
| *Bruggmanniella_duguetiae* |  | 0 | 1 | 0 | 0 | 1 | 1 | 0 | ? | ? | ? | ? | ? | ? | ? | ? | ? | ? | ? | ? | ? | 1 | 2 | 1 | 1 | 1 | 1 |
| *Bruggmanniella_doliocarpi* |  | 0 | 1 | 1 | 0 | 1 | 1 | 0 | 0 | 1 | 1 | 0 | 1 | 0 | 1 | 0 | 0 | 1 | 0 | 0 | 1 | 1 | 1 | 1 | 1 | 1 | 1 |
| *Bruggmanniella_bumeliae* |  | 0 | 1 | 1 | 0 | 1 | 1 | 0 | 0 | 1 | 1 | 0 | 1 | 0 | 1 | 0 | 0 | 1 | 0 | 0 | 1 | 1 | 1 | 1 | 1 | 1 | 1 |
| *Bruggmanniella_maytenuse* |  | 0 | ? | 1 | 0 | 1 | 1 | 0 | 0 | 1 | 1 | 0 | 1 | 0 | 1 | 0 | ? | 1 | 1 | 2 | ? | 1 | 1 | 1 | 1 | 1 | 3 |
| *Bruggmanniella_ingae* |  | 0 | 1 | 1 | 0 | 1 | 1 | 0 | 0 | 1 | 1 | 0 | 1 | 0 | 1 | 0 | 0 | 1 | 1 | 0 | 2 | 1 | 1 | 1 | 1 | 1 | 2 |
| *Bruggmanniella_oblita* |  | 0 | 1 | 1 | 0 | 1 | 0 | 1 | ? | ? | ? | ? | ? | ? | ? | ? | ? | ? | ? | ? | ? | 1 | 1 | 1 | 1 | 1 | 1 |
| *Bruggmanniella_braziliensis* |  | 0 | 1 | 1 | 0 | 1 | 2 | 1 | ? | 1 | 1 | 0 | 1 | 0 | 1 | 0 | 0 | 1 | 1 | 0 | 1 | 1 | 2 | 1 | 1 | 1 | 1 |
