# Supplementary material for: Cladistic analysis of the genus Bruggmanniella Tavares (Diptera, Cecicomyiidae, Asphondyliini) with evolutionary inferences on the gall inducer-host plant association and description of a new Brazilian species
Source: PLoS One. 2020 Feb 5;15(2):e0227853. doi: 10.1371/journal.pone.0227853 (PMC7001989; doi:10.1371/journal.pone.0227853)
Supplement: S1 Fig — (DOCX) [file pone.0227853.s005.docx]

**S5 Figure –** **Tree under equal weight**

Numbers on each branch indicate synapomorphies.
